# A lipid-immune network signature defines susceptibility to asparaginase-associated pancreatitis

**DOI:** 10.1172/jci.insight.202662

**Published:** 2026-04-28

**Authors:** Cheng-Yu Tsai, Na Bo, Thai Hoa Tran, Maisam Abu-El-Haija, Gayathri Swaminathan, Bomi Lee, Sudhir Ghandikota, Li Wen, Yves Théorêt, Steven D. Mittelman, Elena J. Ladas, Anil G. Jegga, Lewis B. Silverman, Ying Ding, Sohail Z. Husain

**Affiliations:** 1 Division of Gastroenterology, Hepatology and Nutrition, Department of Pediatrics, Stanford University, Palo Alto, California, USA.; 2 Department of Biostatistics, Virginia Commonwealth University, Richmond, Virginia, USA.; 3 Division of Pediatric Hematology Oncology, Charles-Bruneau Cancer Center, Centre Hospitalier Universitaire Sainte-Justine, Montreal, Quebec, Canada.; 4 Department of Pediatrics, University of Cincinnati College of Medicine, Cincinnati, Ohio, USA.; 5 Division of Pediatric Gastroenterology and; 6 Division of Biomedical Informatics, Cincinnati Children’s Hospital Medical Center, Cincinnati, Ohio, USA.; 7 Center for Biomarker Discovery and Validation, Peking Union Medical College Hospital, Chinese Academy of Medical Sciences and Peking Union Medical College, Beijing, China.; 8 Département Clinique de Médecine de Laboratoire, Secteur Pharmacologie Clinique, Optilab Montréal, Centre Hospitalier Universitaire Sainte-Justine, Montreal, Canada.; 9 Department of Pediatric Endocrinology, University of California, Los Angeles, California, USA.; 10 Division of Pediatric Hematology, Oncology, and Stem Cell Transplant, Columbia University Irving Medical Center, New York, New York, USA.; 11 Institute of Human Nutrition, Columbia University, New York, New York, USA.; 12 Department of Biostatistics and Health Data Science, University of Pittsburgh, Pittsburgh, Pennsylvania, USA.

**Keywords:** Gastroenterology, Hematology, Oncology, Biomarkers, Lipidomics, Proteomics

## Abstract

**BACKGROUND:**

Asparaginase is essential for curing acute lymphoblastic leukemia (ALL), but its use is limited by asparaginase-associated pancreatitis (AAP), a severe and unpredictable toxicity lacking validated prospective biomarkers. We sought to define early systemic molecular features of susceptibility to AAP.

**METHODS:**

We performed longitudinal lipidomic and proteomic profiling in two independent pediatric ALL cohorts (*n* = 161; 79 AAP cases, 82 controls) using paired blood samples collected before asparaginase exposure and at the end of induction therapy (including a single dose of asparaginase), thereby capturing pre-injury biology rather than consequences of pancreatitis. We applied differential abundance and network-based analyses and integrated lipid-cytokine associations using proteomics.

**RESULTS:**

Across cohorts, we identified a reproducible lysophosphatidylcholine-centered (LPC-centered) signature characterized by attenuated induction therapy–associated LPC responses and disruption of LPC coregulation at the network level. Proteomic profiling revealed enrichment of cytokine signaling pathways, and integrative analyses demonstrated altered lipid-cytokine coupling, including a flip in association direction for LPC species and IL-18 between cases and controls. Although IL-18/LPC ratios did not differ globally, elevated postinduction IL-18/LPC ratios identified AAP risk within a protocol-defined very high-risk ALL subgroup (AUC = 0.81).

**CONCLUSION:**

These findings support a systems-level model in which failure of coordinated lipid-immune responses under therapeutic stress confers vulnerability to AAP, providing a framework for validation and mitigation strategies.

**TRIAL REGISTRATION:**

NCT00400946; NCT01574274; NCT03020030 (parent trials).

**FUNDING:**

Servier Pharmaceuticals (IIT-95014-027-USA); SDRC (P30DK116074); Stanford SPARK; Fonds de Recherche du Québec – Santé; Fondation Charles-Bruneau; Leukemia & Lymphoma Society of Canada.

## Abstract 2

Early blood “omics” patterns revealed disrupted lipid–immune responses linked to dangerous drug-induced pancreatitis in children with leukemia treated with the crucial chemotherapy asparaginase.

## Graphical abstract

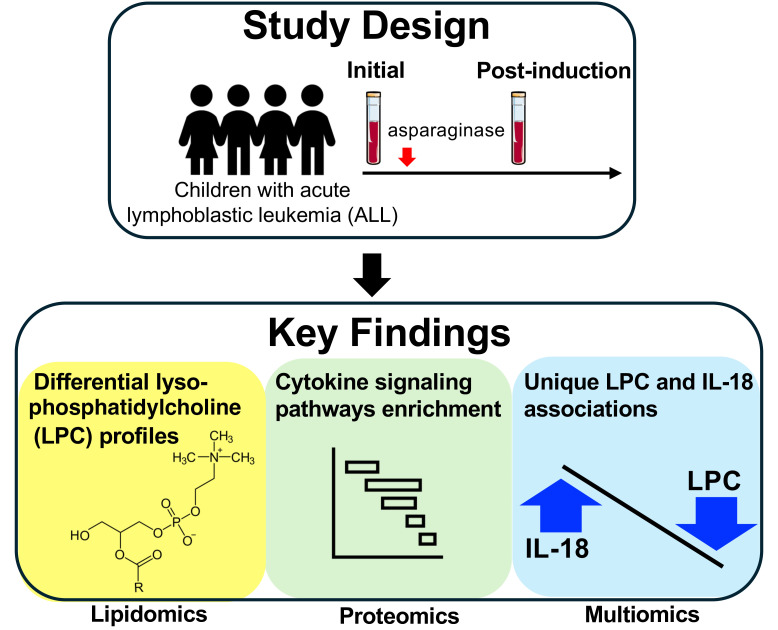

## Introduction

Drug-induced toxicities remain a major cause of morbidity and treatment interruption across medical disciplines, particularly when they arise unpredictably and lack reliable biomarkers of susceptibility (1). Among these, drug-induced pancreatitis represents a uniquely severe complication, associated with substantial acute morbidity, long-term pancreatic sequelae, and mortality risk (2–4). Asparaginase-associated pancreatitis (AAP) exemplifies this challenge. Asparaginase is an indispensable component of therapy for acute lymphoblastic leukemia (ALL), the most common pediatric malignancy, yet AAP develops in approximately 5%–20% of treated patients and frequently necessitates dose reduction or permanent discontinuation of therapy (5–7). These interruptions are clinically consequential, as incomplete asparaginase exposure is associated with inferior leukemia outcomes, and survivors of AAP may experience pancreatic exocrine insufficiency, diabetes, recurrent or chronic pancreatitis, or other serious complications (6, 8–10). Despite its clinical impact, the biological mechanisms underlying AAP remain incompletely understood, and existing clinical and genetic predictors offer only modest discriminative performance (6, 11, 12). Current risk stratification relies largely on demographic and treatment-related factors, providing limited insight into the systemic biological programs that predispose certain patients to pancreatic injury after asparaginase exposure. Critically, there are no validated biomarkers that identify susceptible individuals before clinical pancreatitis emerges, representing a major unmet need for biomarker-guided mitigation strategies for AAP.

Lipids are central regulators of membrane integrity, cellular signaling, and inflammatory responses, and perturbations in lipid metabolism are increasingly recognized as key contributors to inflammatory and metabolic diseases (13, 14). Altered lipidomic landscapes have been described in atherosclerosis (15), nonalcoholic fatty liver disease (16, 17), metabolic syndrome (18), sepsis (19), and cancer (20, 21), often reflecting coordinated disruptions in immune-metabolic signaling rather than isolated metabolic defects. In the context of ALL and its treatment, prior studies have reported substantial lipid alterations in leukemic states, during induction chemotherapy, and in acute pancreatitis of diverse etiologies (22–27). However, a systematic, longitudinal characterization of lipidomic changes that precede AAP onset has not been performed, and it remains unknown whether specific lipid programs mark susceptibility to pancreatic injury during asparaginase therapy.

Importantly, lipid signaling does not operate in isolation. Lipids engage in bidirectional crosstalk with cytokine networks and stress-response pathways that shape inflammatory tone and tissue resilience. Newer proteomic profiling techniques offer a complementary lens through which to interrogate these interactions, particularly for low-abundance cytokines and immune mediators that are poorly captured by conventional mass spectrometry–based approaches (28). Integrating lipidomic and proteomic data therefore provides a powerful strategy to identify coordinated molecular signatures that may underlie differential susceptibility to drug-induced organ injury.

Here, we conducted a nested case control analysis within prospective Dana-Farber Cancer Institute (DFCI) ALL clinical trials, integrating a multiomic approach to identify early systemic molecular signatures associated with subsequent development of AAP. Leveraging longitudinal plasma and serum samples collected before and after asparaginase-containing induction therapy, we performed large-scale lipidomic profiling across 2 independent pediatric ALL cohorts and integrated these data with high-fidelity proteomic measurements of inflammatory and signaling pathways. By anchoring our analyses to samples obtained prior to clinical pancreatitis, we sought to identify molecular perturbations that emerge during induction and precede overt pancreatic injury. Through this integrated framework, we uncover a reproducible, lysophosphatidylcholine-centered (LPC-centered) lipid profile and reveal a pathogenic shift in LPC-cytokine relationships. Specifically, we identify an LPC to IL-18 signature that is associated with AAP within a protocol-defined very high-risk (VHR) ALL subgroup. These findings provide mechanistic insight into AAP susceptibility and establish a foundation for future biomarker development and prospective validation to support AAP risk stratification.

## Results

### Prospective lipidomic profiling identifies postinduction lipid alterations preceding AAP.

**Figure 1:**
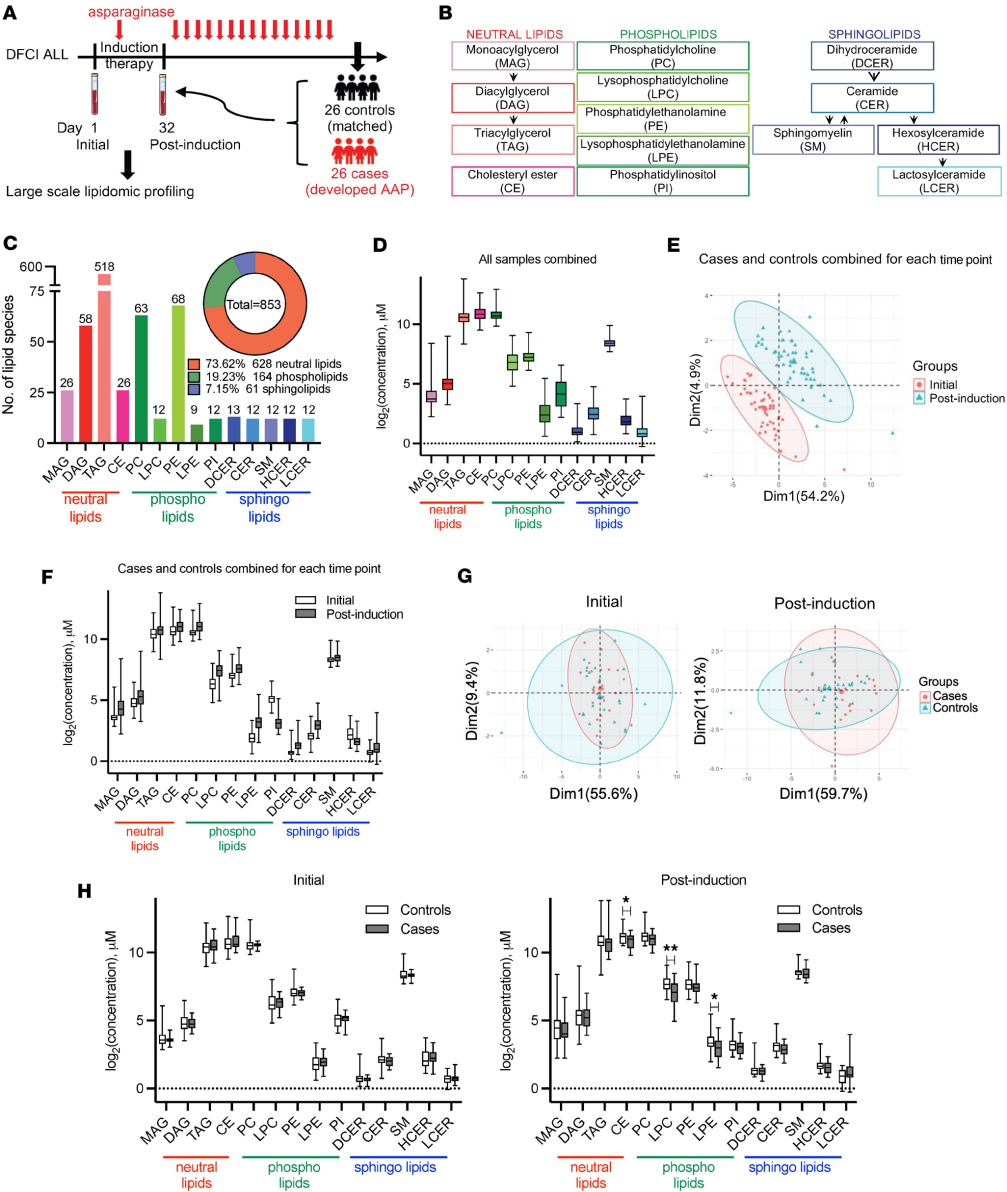
Global lipidomic landscape across induction therapy in the discovery cohort. (**A**) Study schematic for the discovery cohort, showing prospective plasma sample collection at 2 time points: an initial pre-asparaginase time point and a postinduction time point following exposure to asparaginase-containing induction therapy. (**B**) Overview of the plasma lipidome measured using the Metabolon Complex Lipid Panel, comprising 3 major lipid groups (neutral lipids, phospholipids, sphingolipids) and 14 lipid classes. (**C**) Number and proportion of lipid species detected within each lipid class and group after quality filtering. (**D**) Box-and-whisker plots showing the distribution of lipid class concentrations across all samples, demonstrating expected dominance of cholesteryl esters, triacylglycerols, phosphatidylcholines, and sphingomyelins in human plasma. (**E**) PCA illustrating a strong separation between initial and postinduction samples when cases and controls are combined, indicating a global lipidomic shift during induction therapy. (**F**) Comparison of lipid class concentrations between initial and postinduction time points, highlighting increased dispersion and dynamic range postinduction. (**G**) PCA stratified by case-control status at each time point shows minimal separation at baseline but modest divergence postinduction. (**H**) Lipid class–level comparisons between cases and controls reveal selective postinduction reductions in lysophosphatidylcholine (LPC), lysophosphatidylethanolamine (LPE), and cholesteryl ester (CE) among cases. *P < 0.05, **P < 0.01; unpaired 2-tailed *t* test with Welch’s correction. The discovery cohort includes 26 cases and 26 controls. Unless otherwise indicated, analyses include all available samples from these participants at each time point. For all box-and-whisker plots (**D**, **F**, and **H**), center line = median; box = IQR; whiskers = min–max.

To identify systemic lipid perturbations that precede the development of AAP, we first performed comprehensive lipidomic profiling in a prospectively sampled discovery cohort of 52 children and adolescents enrolled in DFCI clinical trials (protocols 05-001, ref. 29; 11-001, ref. 30). Plasma samples were collected at 2 time points: an initial sample obtained early during induction therapy prior to any asparaginase exposure (specifically on day 1 for DFCI 05-001 and day 7 for DFCI 11-001), and a postinduction sample collected at the completion of induction after a single asparaginase dose (day 32; Figure 1A). This initial time point preceded asparaginase exposure but did not necessarily precede exposure to other induction agents, including corticosteroids and vincristine (25, 29, 30). Baseline demographic and clinical characteristics of cases and controls have been previous published (31) and are summarized in Tables 1 and 2. Overall characteristics were similar between cases and controls. Primary models were additionally adjusted for prespecified covariates (age, BMI, and ALL risk group; see Methods). Among 52 study participants, 26 developed pancreatitis within 9 months of induction (cases); 26 controls, matched to sex, age, asparaginase formulations, and initial ALL risk, did not. The timing of pancreatitis onset among these cases, including 5 participants who developed pancreatitis during induction therapy (early cases), has been reported (31).

Using the Metabolon Complex Lipid Panel, we detected 944 lipid species spanning 3 major lipid groups and 14 lipid classes, with 853 species retained for downstream analysis after quality filtering (Figure 1, B and C, and Supplemental Table 1; supplemental material available online with this article; https://doi.org/10.1172/jci.insight.202662DS1). The overall plasma lipidome composition was consistent with established reference profiles, with neutral lipids predominating, followed by phospholipids and sphingolipids (Figure 1D) (14, 32–34).

The biostatistical analysis methods used to assess the lipidomic profiles between cases and controls at each of the 2 time points are detailed in Supplemental Table 2. Principal component analysis (PCA) revealed clear separation between initial and postinduction samples when cases and controls were combined, indicating a global remodeling of the lipidome during induction therapy (Figure 1E). This shift likely reflects the combined metabolic effects of induction chemotherapy, including corticosteroids and asparaginase, and was accompanied by increased lipid class variability after induction (Figure 1F) (25, 35). At each time point, the cases and controls largely overlapped in PCA (Figure 1G). At the initial time point, lipid class concentrations were comparable (Figure 1H), suggesting similar baseline lipid states prior to asparaginase exposure. By contrast, postinduction comparisons revealed selective lipid class differences between cases and controls (Figure 1H). Notably, LPC, lysophosphatidylethanolamine (LPE), and cholesteryl esters were reduced in cases relative to controls after induction, implicating specific lipid pathways rather than global lipid depletion.

### Linear mixed-effects modeling identifies LPC as the dominant lipid class differentiating AAP cases.

**Figure 2:**
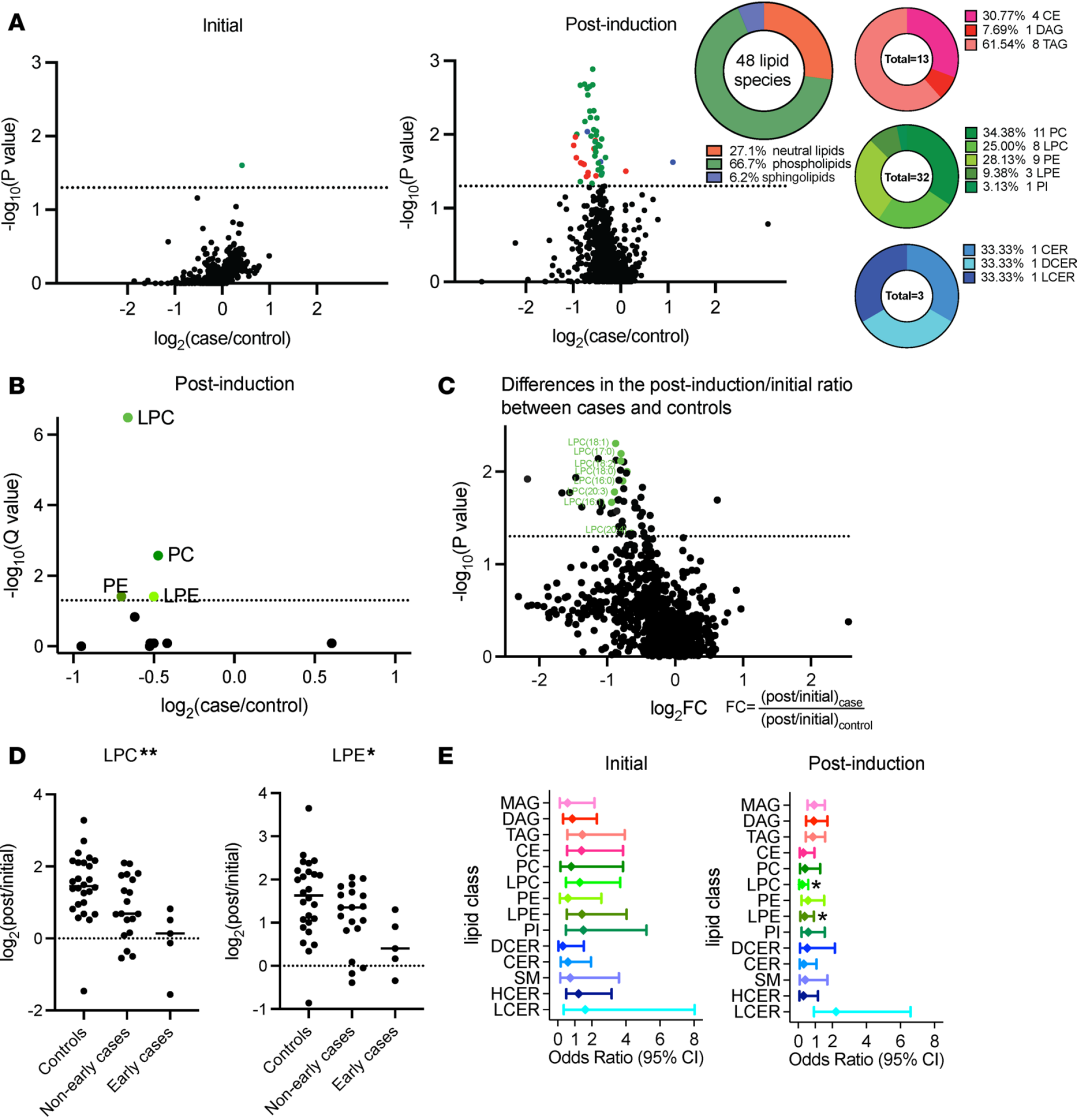
Linear mixed-effects modeling identifies LPC species as key differentiators of AAP cases. (**A**) Differential lipid species abundance between cases and controls at each time point using linear mixed-effects models adjusted for age, BMI, and initial ALL risk. After induction, 48 lipid species are significantly different, predominantly phospholipids. (**B**) Lipid class enrichment analysis of the 48 postinduction differential lipid species demonstrates strong overrepresentation of LPC, followed by PC, PE, and LPE. *q* < 0.05, Fisher’s exact test. (**C**) Within-subject postinduction to initial ratios reveal that 8 LPC species show significantly attenuated induction-associated increases in cases (*n* = 24) compared with controls (*n* = 26). (**D**) Composite LPC and LPE postinduction/initial ratios stratified by timing of pancreatitis onset show a graded pattern: highest in controls (*n* = 26), intermediate in non-early cases (*n* = 19), and lowest in early AAP cases (*n* = 5). *P < 0.05, **P < 0.01, linear trend test. (**E**) Multivariable logistic regression demonstrates that higher postinduction (26 controls and 24 cases) LPC and LPE concentrations are independently associated with reduced AAP risk. ORs are shown per 1-unit increase in log_2_ lipid concentration, adjusted for age, BMI, and initial ALL risk. *95% CI does not cross 1. The discovery cohort includes 26 cases and 26 controls. Unless otherwise indicated, analyses include all available samples from these participants at each time point.

To rigorously identify lipid species associated with subsequent AAP, we applied linear mixed-effects models incorporating both time points and adjusting for age, BMI, and initial ALL risk (31). At the initial time point, only a single lipid species differed between cases and controls (phosphatidylinositol [PI]) (18:1/18:2); Figure 2A), but this did not remain significant after multiple-testing correction by FDR, and the PI class was not significant in class-level modeling (Supplemental Table 3), reinforcing the absence of baseline differences.

In contrast, postinduction modeling identified 48 lipid species that differed significantly between cases and controls (*P* < 0.05), with 46 species reduced in cases (Figure 2A and Supplemental Table 4). These species clustered disproportionately within phospholipid classes, particularly LPC (*q* < 0.0001), followed by phosphatidylcholine (PC), phosphatidylethanolamine (PE), and LPE (Figure 2B). Eight LPC species — LPC(16:0), LPC(16:1), LPC(17:0), LPC(18:0), LPC(18:1), LPC(18:2), LPC(20:3), and LPC(20:4) — accounted for the strongest and most consistent differences.

To distinguish absolute concentration differences from therapy-induced dynamics, we compared within-subject postinduction-to-initial ratios. All 8 LPC species exhibited significantly attenuated post/initial increases in cases relative to controls (Figure 2C and Supplemental Figure 1), indicating a failure to mount a normal LPC response during induction rather than baseline deficiency. Although several individual neutral lipid species (predominantly triacylglycerol [TAG] and diacylglycerol [DAG]) exhibited relatively large absolute log_2_ fold-change values, these represented a small fraction of the measured species within those classes (e.g., TAG: 11/518, 2.1%; DAG: 2/58, 3.4%) and did not reflect a coordinated class-level shift. Consistent with this pattern, class-level modeling corroborated these findings. Although no lipid class differed at baseline, postinduction LPC and LPE concentrations were significantly lower in cases, and the LPC post/initial ratio remained reduced after covariate adjustment (Supplemental Table 3). Further stratification by pancreatitis timing, using a schema applied in previous omics studies of AAP (31), revealed a graded relationship: controls exhibited the greatest LPC increase, non-early cases showed intermediate responses, and early AAP cases displayed the lowest LPC and LPE ratios (Figure 2D and Supplemental Figure 2). This graded pattern was consistent across the 8 LPC species identified in Figure 2C (Supplemental Figure 3). Interestingly, this graded pattern for LPC and LPE mirrors our prior metabolomic observations in the same cohort (31), in which early AAP cases exhibited the lowest postinduction ratios of vitamin A metabolites, including retinol and carotenoids. Together, these findings suggest that early susceptibility to AAP may reflect broader perturbations in lipid and metabolite homeostasis rather than isolated abnormalities in a single pathway.

Multivariable logistic regression demonstrated that higher postinduction LPC concentrations were associated with a substantially reduced odds of AAP (OR 0.22, 95% CI 0.06–0.58), with LPE showing a weaker association (OR 0.38, 95% CI 0.13–0.91; Figure 2E). In species-level analyses at the postinduction time point (Supplemental Figure 4), LPC(18:0) showed the strongest inverse association with AAP (OR 0.21, 95% CI 0.06–0.58). Multiple additional LPC species also had 95% CIs not crossing 1, indicating that the class-level LPC association is not attributable to a single species. Across analytic approaches, LPC emerged as the most robust lipid class associated with AAP susceptibility.

### Network analysis reveals disruption of LPC coregulation preceding and following induction.

To determine whether AAP was associated not only with altered lipid abundance but also with disrupted lipid coordination, we applied weighted gene coexpression network analysis (WGCNA) in a data-driven manner (36, 37). At the initial time point, lipid species clustered into 11 modules based on correlated abundance patterns in controls (Supplemental Figure 5A). Module preservation analysis identified 3 modules that were not preserved in cases, indicating altered correlation structure prior to asparaginase exposure (Supplemental Figure 5B and Supplemental Table 5). Strikingly, 11 of 12 detected LPC species localized to a single nonpreserved module (M11, Supplemental Figure 5), including all 8 LPC species identified in the differential abundance analysis (Figure 2C and Supplemental Table 4). These findings indicate that LPC dysregulation in AAP is detectable at the network level even before treatment-induced lipid shifts occur. Postinduction WGCNA revealed further divergence. We also performed postinduction module preservation analysis in the discovery cohort, and the evaluable modules were preserved. Because uncorrelated lipid species are excluded from preservation calculations, we additionally examined changes in the uncorrelated lipid species set. The proportion of uncorrelated lipid species increased markedly in cases, particularly among phospholipids and sphingolipids (Supplemental Figure 6 and Supplemental Table 6). Notably, 11 of 12 LPC species were classified as uncorrelated in cases after induction, consistent with widespread disruption of LPC coregulation during therapy. The absence of module membership among these lipids (i.e., uncorrelated lipid species) indicates loss of coordinated regulation rather than stochastic variation, consistent with biologically meaningful disruption of LPC-centered metabolic networks during induction therapy (36).

### Independent validation cohort recapitulates LPC-centered network disruption.

**Figure 3:**
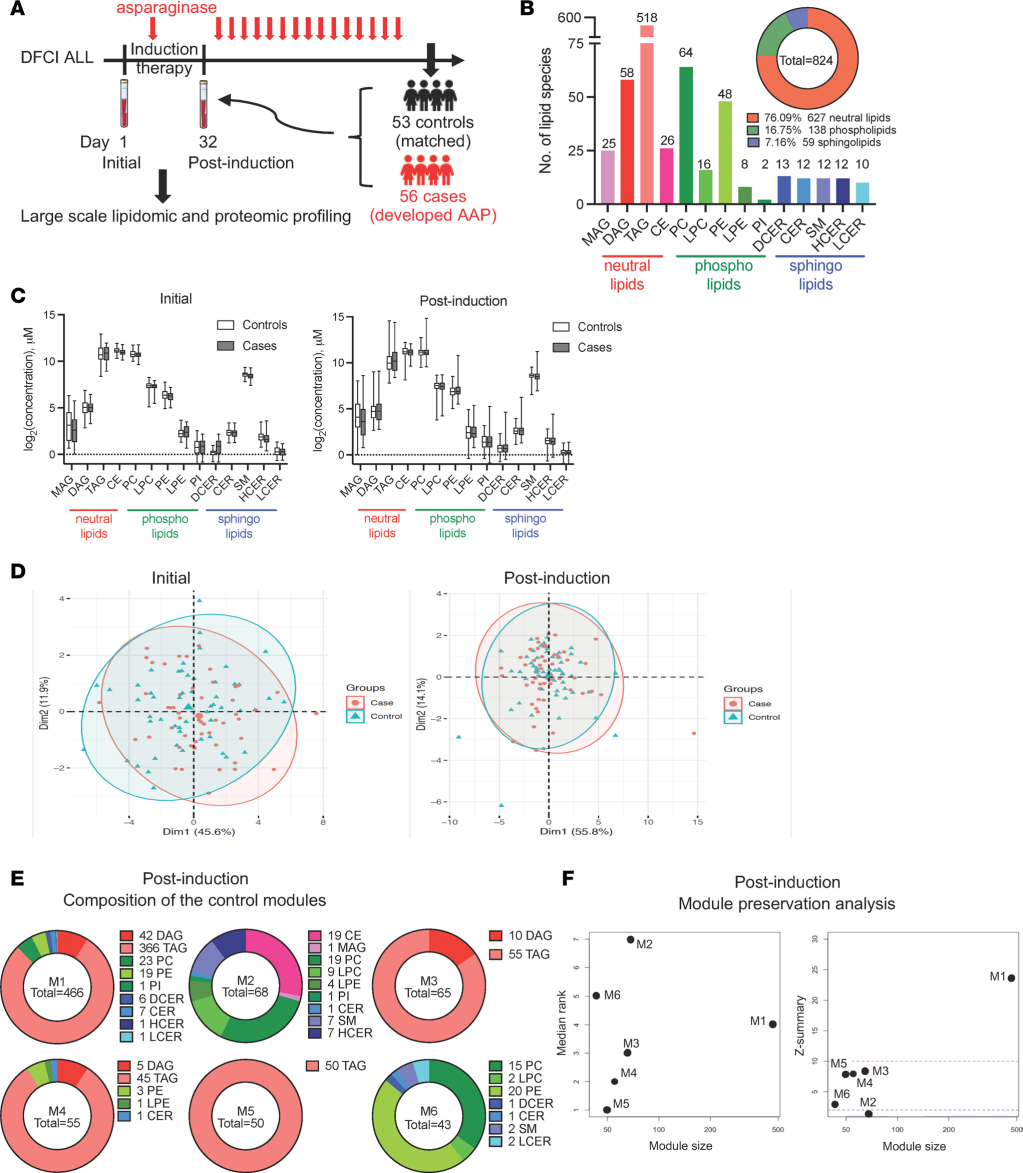
LPC-centered lipidomic signatures replicate in an independent validation cohort. (**A**) Study schematic for the independent validation cohort, showing identical longitudinal sampling design. (**B**) Distribution of lipid species across lipid classes mirrors that of the discovery cohort, with more than 90% overlap in detected species. (**C**) Lipid class concentrations across time points demonstrate similar induction-associated shifts as observed in the discovery cohort. Box-and-whisker plots show median (center line), IQR (box), and range (whiskers: min–max). (**D**) PCA stratified by case-control status shows limited global separation, emphasizing the need for network-level analyses. (**E**) WGCNA modules identified in postinduction samples from controls, ranked by module size. (**F**) Module preservation analysis identifies a nonpreserved module enriched for LPC species in cases, indicating disrupted LPC coregulation after induction therapy. Validation cohort includes 56 cases and 53 controls. Unless otherwise indicated, analyses include all available samples from these participants at each time point.

To validate these findings, we analyzed an independent cohort of 109 pediatric ALL patients enrolled in DFCI 16-001 (53 AAP cases, 56 controls) (31). Baseline demographic and clinical characteristics of cases and controls are summarized in Tables 3 and 4. Lipidomic coverage and class distributions closely mirrored those of the discovery cohort, with greater than 90% overlap in detected species (Figure 3, A–C, and Supplemental Table 7). Unlike the discovery cohort, global PCA separation between cases and controls showed substantial overlap (Figure 3D), and we did not detect statistically significant case-control differences using linear mixed-effects or multivariable models. This could reflect differences between the validation and discovery cohorts, including nonfasting collection influenced by regional diet, serum versus plasma matrix effects, and clinical trial protocol differences, which can increase variability and obscure mean shifts that these models are designed to detect. We therefore next applied network-based analysis. At the initial time point, WGCNA categorized 10 of 16 LPCs (62.5%) in controls as uncorrelated lipid species. Consistent with this, 35.3% of phospholipids (including LPCs) and 63.7% of sphingolipids were uncorrelated and therefore excluded from preservation testing, making module preservation analysis less informative at this time point; the evaluable modules were preserved. In contrast, postinduction WGCNA identified a nonpreserved module (M2) enriched for LPC species when controls were used as the reference (Figure 3, E–F, and Supplemental Table 8). The 8 LPC species previously identified in the discovery cohort localized to nonpreserved or weakly preserved modules (M2 and M6) in the validation cohort, confirming reproducible LPC-centered network disruption across independent populations. Together, these cross-cohort analyses support LPC dysregulation as a consistent feature of AAP susceptibility.

### Proteomic profiling reveals cytokine pathway enrichment.

To investigate whether lipid alterations were accompanied by coordinated changes in circulating proteins, we performed serum proteomic profiling in the validation cohort using the Olink Explore 3072 platform. Proteomic profiling was restricted to the validation cohort because insufficient plasma from the discovery cohort was available for proteomics. Of 2,943 proteins assayed, 2,538 passed quality filters and were included in downstream analyses for each time point (Supplemental Figure 7 and Supplemental Table 9).

**Figure 4:**
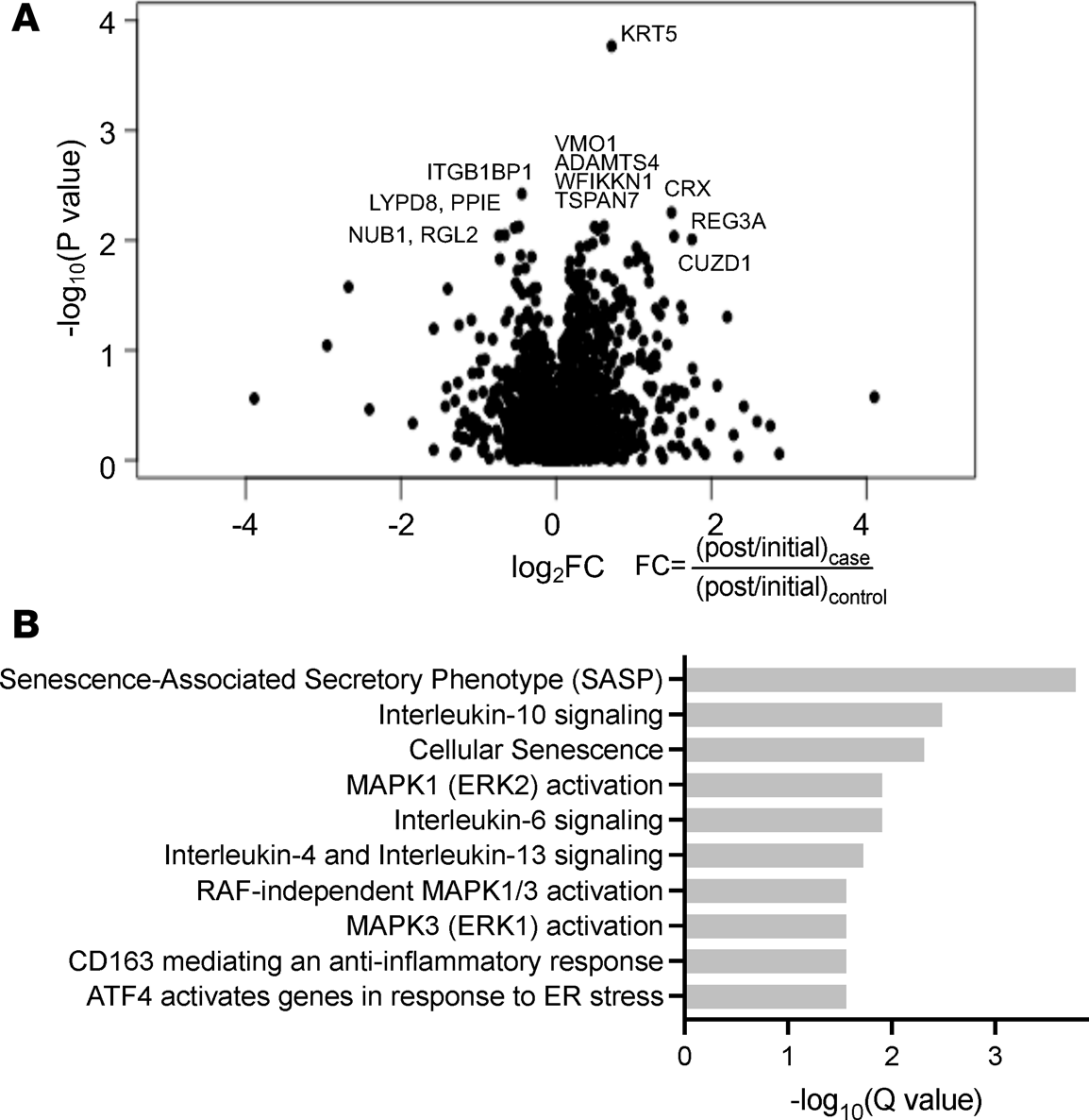
Proteomic profiling reveals cytokine pathway enrichment associated with AAP. (**A**) Volcano plot showing differentially expressed proteins based on within-subject postinduction-to-initial ratios between cases and controls. Only proteins with a *P* value less than 0.01 were annotated. (**B**) Pathway enrichment analysis demonstrates that all top enriched pathways are cytokine-related, including pathways previously implicated in pancreatitis-associated inflammation. *q* < 0.05, Fisher’s exact test. Validation cohort proteomics: controls, *n* = 48; cases, *n* = 52.

Within-subject post/initial comparisons identified 103 differentially expressed proteins between cases and controls (Figure 4A). Pathway enrichment analysis revealed that all 10 significantly enriched pathways were cytokine-related (Figure 4B and Supplemental Table 10), including IL-6 signaling and other inflammatory cascades previously implicated in acute pancreatitis (38, 39).

**Figure 5:**
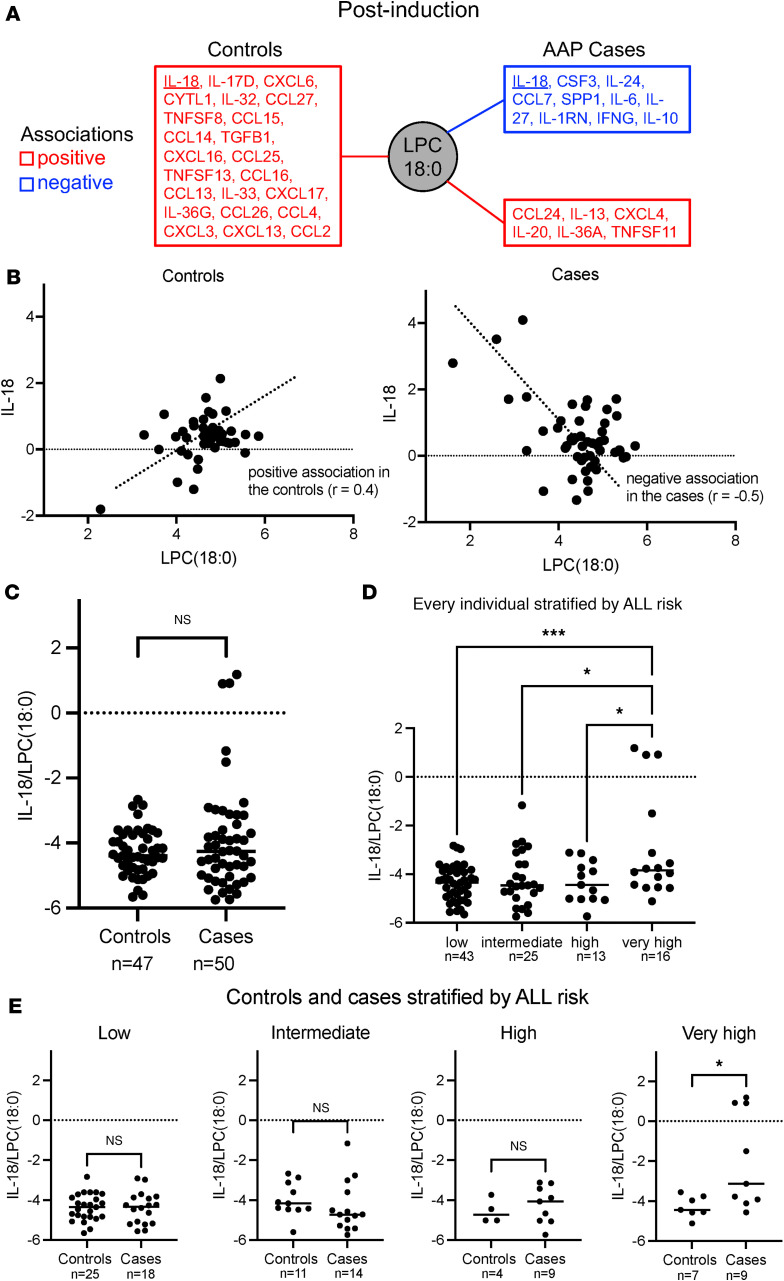
Integrated lipidomic-proteomic analysis identifies an IL-18/LPC signature in VHR ALL. (**A**) Network visualization of LPC(18:0)-cytokine associations show predominantly positive associations in controls and a mixed pattern in cases, with IL-18 switching from a positive association in controls to a negative association in cases. (**B**) Pearson’s correlation analysis confirms a positive IL-18–LPC(18:0) relationship in controls and an inverse relationship in cases. Both axes are presented on a log_2_ scale. (**C**) IL-18/LPC(18:0) ratios do not differ globally between cases and controls but reveal a subset of cases with markedly elevated ratios. Unpaired 2-tailed *t* test with Welch’s correction. (**D**) Stratification by ALL risk demonstrates significantly higher IL-18/LPC(18:0) ratios in participants with VHR ALL. *P < 0.05, ***P < 0.001; 1-way ANOVA. (**E**) Within the VHR subgroup, cases show significantly elevated IL-18/LPC(18:0) ratios compared with controls. *P < 0.05; unpaired 2-tailed *t* test with Welch’s correction.

Given the convergence of lipid and cytokine signals, we examined postinduction associations for each of the 8 significant LPC species and 109 cytokines (Supplemental Table 11). The complete LPC-cytokine association matrix is shown in Supplemental Figure 8. Among the 8 LPC species, LPC(18:0) showed the greatest case-control divergence: in controls, LPC(18:0) was positively associated with 23 cytokines, whereas in cases it showed fewer positive and more negative associations (Figure 5A).

### IL-18/LPC ratios reveal a context-dependent AAP susceptibility signal in VHR ALL.

Notably, the association between LPC(18:0) and IL-18 switched direction, from positive in controls to negative in cases (Figure 5, A and B). Although absolute IL-18/LPC(18:0) ratios did not differ globally between cases and controls (Figure 5C), stratification by ALL risk revealed that this ratio was selectively elevated among participants with VHR ALL (Figure 5D). In DFCI 16-001, VHR ALL is protocol-defined by adverse leukemia biology (including IKZF1 deletion) and/or by persistently elevated minimal residual disease at the end of Induction 1B, assessed approximately 10 weeks after starting therapy (40). Together, these observations suggest that the IL-18/LPC imbalance is most apparent within a biologically high-risk leukemia context.

When cases and controls were examined within each DFCI 16-001 risk stratum, the IL-18/LPC(18:0) ratio was significantly elevated in cases compared with controls specifically within the VHR subgroup, but not within the low-, intermediate-, or high-risk groups (Figure 5E). Consistent with this risk-restricted pattern, receiver operating characteristic (ROC) analysis limited to the VHR subgroup showed discrimination of AAP cases from controls (AUC = 0.81; *P* < 0.05) (Supplemental Figure 9).

A parallel pattern was observed for IL-18/LPC(20:4), which was identified in the same panel-wide analysis of all 8 LPC species and exhibited similar case-control divergence within the VHR subgroup (Supplemental Figure 10), supporting the robustness of an IL-18/LPC axis rather than dependence on a single lipid species. Collectively, these findings indicate that IL-18/LPC ratios provide context-dependent discriminatory information, particularly within the VHR ALL population. Clinical characteristics of the VHR subgroup are summarized in Supplemental Table 12.

To explore whether this VHR-restricted IL-18/LPC signal aligns with underlying leukemia biology, we examined available leukemic-cell transcriptomic data. RNA-Seq data were available for 10 of 16 VHR participants (3 controls and 7 cases) (40). Among the 7 VHR cases who developed AAP, 4 exhibited Philadelphia chromosome–like (Ph-like) ALL, characterized by kinase-activating lesions involving the JAK/STAT pathway (ZBTB44:JAK2, GOLGB1:JAK2, IL7R L243>PSCS, SH2B3 F146fs) or ABL-class fusions (ETV6:ABL1), whereas none of the controls demonstrated Ph-like features. In addition, 4 of 7 VHR cases harbored RAS/MAPK pathway mutations (KRAS G12D, KRAS A59E, KRAS A59G, FLT3 N676S), which were absent in the controls.

Although these transcriptomic observations are descriptive and limited by sample size, they suggest that the IL-18/LPC imbalance associated with AAP may preferentially arise in leukemias marked by kinase-driven signaling programs and heightened inflammatory or metabolic stress. Further studies will be required to determine whether IL-18/LPC profiles associated with AAP are consistently enriched within specific ALL molecular subtypes, particularly those with kinase-activating alterations such as Ph-like ALL.

## Discussion

In this study, we performed a nested case control analysis within 3 prospective DFCI ALL clinical trials to define systemic molecular signatures associated with susceptibility to AAP using a multiomic approach. The discovery cohort was derived from 2 clinical trial protocols, and the validation cohort was drawn from an independent third protocol. By integrating lipidomics, network-based analyses, and high-dimensional proteomics across 2 independent pediatric ALL cohorts and by anchoring analyses to samples collected prior to overt pancreatic injury, we identified a reproducible, LPC-centered susceptibility signature that emerges during induction therapy. This signature is characterized not only by altered LPC abundance and dynamics, but also by disruption of LPC coregulation and altered coupling to inflammatory cytokine pathways, including a context-dependent association with IL-18. Together, these findings support a model in which AAP susceptibility reflects a failure of coordinated lipid–immune stress responses rather than a single static biomarker abnormality.

AAP has long been recognized as a clinically consequential toxicity of asparaginase therapy, yet mechanistic understanding of why only a subset of patients develops pancreatitis remains limited. Prior work has largely focused on demographic risk factors, treatment variables, and selected genetic associations, which collectively provide incomplete predictive power and limited biological insight (6, 7, 9, 11, 12, 41–45). Our findings shift the conceptual framework from one of isolated risk factors to one of system-level vulnerability. Specifically, we show that induction therapy imposes a global metabolic perturbation, but that individuals who develop AAP fail to maintain coordinated phospholipid responses, most prominently involving LPC, during this stress. This failure is detectable at multiple analytic levels and precedes clinical pancreatitis, suggesting that susceptibility is encoded in how biological systems adapt to therapy rather than in baseline lipid levels alone.

Across species-level differential abundance testing, lipid class enrichment, longitudinal ratio analyses, timing-based stratification, and risk modeling, LPC emerged as the most consistent lipid class associated with AAP. Importantly, LPC dysregulation was not limited to reduced postinduction concentrations; it was also characterized by attenuated induction-associated increases and by graded differences corresponding to the timing of pancreatitis onset. These convergent observations argue against a nonspecific depletion effect and instead support a model in which LPC participates in adaptive responses to induction therapy. LPCs are known to influence membrane integrity, lipid signaling, immune cell recruitment, and inflammatory tone, making them plausible mediators of tissue resilience under metabolic stress (46–61). The consistency of LPC signals across analytic approaches strengthens confidence that this finding reflects underlying biology rather than statistical artifact. Accordingly, mechanistic preclinical studies that modulate the LPC axis — including LPC(18:0) and/or multi-species LPC formulations — before and after asparaginase exposure will help define the biological mechanisms linking LPC regulation to AAP susceptibility.

A key advance this study makes is the use of network-based analyses to interrogate lipid regulation beyond mean abundance. WGCNA revealed that LPC species exhibit altered coregulation in cases both prior to and after induction therapy. At baseline, LPCs clustered within a nonpreserved module in cases, indicating altered correlation structure even before asparaginase exposure. After induction, LPC species became predominantly uncorrelated in cases, reflecting widespread loss of coordinated regulation. These findings are mechanistically meaningful: loss of co-regulation implies disruption of shared regulatory or metabolic pathways, which may render tissues less able to respond to subsequent inflammatory or metabolic insults. Importantly, such network disruption can occur even when mean lipid concentrations differ modestly, underscoring why network-level analyses are essential for understanding susceptibility phenotypes in heterogeneous human populations.

In the independent validation cohort, we observed limited PCA separation and no statistically significant differential abundance by linear mixed-effects or multivariable models. These findings were stated transparently and not minimized. However, mean-based tests are inherently sensitive to biological heterogeneity, sample-type differences (serum versus plasma), and reduced effect sizes, particularly in pediatric cohorts with complex treatment regimens. For this reason, replication in this study was defined not by identical marginal *P* values, but by recurrence of an LPC-centered signature across analytic layers, particularly at the level of correlation structure and species identity. Consistent with this framework, WGCNA in the validation cohort again identified nonpreserved modules enriched for the same LPC species identified in the discovery cohort. This reproducible disruption of LPC network architecture across independent cohorts provides strong evidence that the observed LPC dysregulation reflects a conserved biological phenomenon rather than cohort-specific noise.

Proteomic profiling revealed that proteins differing between cases and controls during induction were enriched almost exclusively in cytokine signaling pathways. This convergence on inflammatory biology provides an important mechanistic bridge between lipid dysregulation and pancreatic injury. Acute pancreatitis is characterized by dysregulated cytokine cascades that amplify local and systemic inflammation (62). Our data suggest that altered lipid regulation, particularly involving LPCs, is coupled to aberrant cytokine signaling during induction therapy, potentially lowering the threshold for pancreatic injury. Notably, this convergence was observed using independent platforms, reinforcing the biological coherence of the multiomic findings.

Among cytokines, IL-18 emerged as a particularly informative node within the LPC-cytokine network. IL-18 was not selected a priori, but rather emerged through differential LPC-cytokine association patterns and a striking direction-switch in its relationship with LPC(18:0) between cases and controls. IL-18 is a well-characterized inflammasome-associated cytokine implicated in stress responses, metabolic inflammation, and pancreatitis severity (63, 64). The observed switch from positive to negative association with LPC in cases suggests a dysregulated feedback relationship rather than a simple elevation or suppression of IL-18 levels. Importantly, this association was context-dependent and not uniform across all study participants, reinforcing the notion that IL-18/LPC interactions reflect susceptibility states rather than universal biomarkers.

The enrichment of IL-18/LPC ratio abnormalities within the VHR ALL subgroup is biologically plausible and should not be viewed as a purely post hoc observation. VHR ALL is defined by adverse leukemia biology, higher treatment intensity, and increased systemic stress. These features plausibly interact with lipid and immune regulatory pathways, amplifying susceptibility to organ injury. Within this subgroup, IL-18/LPC ratios discriminated AAP risk with reasonable performance, suggesting that extreme biological stress may unmask otherwise subtle susceptibility signals. Although these findings require prospective validation, they underscore the importance of biological context when interpreting biomarker behavior.

These findings build on prior work demonstrating associations between lipid-related metabolites, including retinoids and carotenoids, and AAP risk (31). By extending analyses to comprehensive lipidomics, network architecture, and cytokine coupling, the present study reveals dimensions of susceptibility that could not be captured by single-metabolite analyses alone. More broadly, our results align with emerging evidence that metabolic-inflammatory network disruption underlies susceptibility to diverse forms of drug-induced and stress-related organ injury (65). The prospective, pre-injury design of this study uniquely positions it to disentangle susceptibility from consequence.

The LPC-centered signatures and IL-18/LPC ratios identified here should be viewed as candidate susceptibility markers rather than clinically actionable tests. Their primary value lies in defining biological pathways and informing future mechanistic and translational studies. Prospective validation in larger cohorts, evaluation across treatment regimens, and integration with genetic and clinical risk factors will be required before clinical implementation can be considered. In addition, although the breadth of lipidomic and proteomic profiling and integrative network modeling strengthens biological inference, this methodological complexity is not intended for routine clinical implementation. A key next step will be translation of these discovery findings into parsimonious, clinically feasible assays. For example, this could involve targeted liquid chromatography–tandem mass spectrometry quantification of a limited set of prioritized LPC species, potentially combined with immunoassay-based measurement of selected cytokines (including IL-18) or simple ratios, supported by standardized preanalytical handling and rigorous evaluation of clinical performance and decision impact. Importantly, we did not define a single clinical cutoff or report positive and negative predictive values for IL-18/LPC. Given the small number of VHR participants (the subgroup in which IL-18/LPC shows the strongest apparent separation), any data-driven threshold would be statistically unstable and at high risk of overfitting, limiting generalizability. Accordingly, we present these findings as exploratory; robust cutoff selection and reliable estimation of positive and negative predictive values will require larger, prospectively collected VHR cohorts and external validation. Nonetheless, these findings suggest that multiomic, network-aware approaches may enable earlier identification of patients at risk for severe therapy-related toxicities.

This study has several limitations. The observational design precludes causal inference. Sample sizes, particularly within subgroups, are modest, and biospecimen types differed between cohorts. The key findings in this study were identified after initial exposure to asparaginase but prior to the clinical onset of pancreatitis and therefore reflect AAP risk status in the postexposure, predisease period rather than true preexposure predictors. Treatment heterogeneity and unmeasured confounders may also influence lipid and cytokine profiles. These limitations are inherent to human translational studies but are offset by the prospective design, independent validation, and convergence across analytic layers. Future work incorporating experimental models, serial sampling beyond induction, and interventional strategies targeting lipid-immune pathways will be essential to test causality and therapeutic relevance.

In summary, this study provides a biologically integrated, human-based framework for understanding susceptibility to AAP. By demonstrating that AAP risk is linked to disrupted LPC regulation, altered lipid network architecture, and context-dependent cytokine coupling during induction therapy, our findings move beyond descriptive associations toward a systems-level model of treatment-related organ injury. This work highlights the value of integrating multiomic data with network biology to uncover latent vulnerability states that may ultimately inform safer, more individualized cancer therapy.

## Methods

### Sex as a biological variable.

Both male and female participants were included. Cases and controls were matched on sex to ensure comparable sex distributions between groups.

### Study design.

The objective of the case-control lipidomic screening was to investigate the early changes in plasma lipids in participants with ALL who developed pancreatitis after asparaginase treatment. Sample sizes were calculated by power analysis, with considerations for the FDR using the R package (ssize.fdr). We assumed the following: a normal distribution for the differential effects (log_2_-transformed) between cases and controls, with a mean of 1.3 and SD of 1; variance of lipids modeled by an inverse γ (3,1) distribution; approximately 5% of total lipids being differential; and an FDR threshold set at *q* < 0.1. These parameters determined that a sample size of 50 would achieve 80% power. The study design for the case-control lipidomics and proteomics screen adhered to the STROBE guideline (66).

### Study participant characteristics (demographics).

Age, BMI, sex, and ALL initial and final risk group subcategorization were obtained from deidentified DFCI protocol databases. Demographic classifications were analyzed as recorded in the source databases, and investigators did not reclassify participants. ALL risk groups were assigned by the study clinical investigators using protocol-defined criteria.

### Risk group stratification (DFCI ALL protocols).

Risk-group assignment was determined by the treating DFCI ALL protocol (05-001, 11-001, or 16-001) using protocol-defined criteria (29, 30, 40). Briefly, stratification was based on diagnostic clinical and disease features (e.g., age at diagnosis, presenting WBC count, immunophenotype/cytogenetic risk features) and may be refined by early treatment response metrics specified by each protocol (e.g., response to induction therapy and minimal residual disease when available). We used the protocol-assigned risk group recorded in the clinical trial database for all analyses. Detailed stratification algorithms are provided in the published DFCI protocol reports; key criteria used for assignment are summarized in the footnotes for Tables 1–4, and risk-group distributions for cases and controls are summarized in Tables 1 and 2 (discovery cohort) and Tables 3 and 4 (validation cohort).

### Asparaginase formulations in the DFCI 05-001, 11-001, and 16-001 protocols.

Four formulations of asparaginase were utilized in the DFCI 05-001 and DFCI 11-001 protocols from which the participants in the discovery cohort were derived: the native *E*. *coli* L-asparaginase (Elspar, Merck; discontinued in the United States since December 2012); the long-acting and most commonly used pegylated form (pegaspargase, Oncaspar, Servier); the newer and longer-acting pegylated formulation (calaspargase pegol, Asparlas, Servier); and the less commonly used Erwinia-derived form (Erwinaze, Jazz Pharm). A detailed breakdown of the participants by protocol and the types of asparaginase they received has been previously published (31). Participants in the external validation cohort (DFCI 16-001 protocol) exclusively received pegaspargase.

### Blood sample collection and preparation.

All participants provided consent to donate additional blood for research purposes. The procedures for sample collection and preparation are detailed in our previous study (25, 31). In the discovery cohort, deidentified plasma samples were collected in a nonfasting state from each participant recruited from the DFCI parent site in Boston and frozen within 24 hours of collection. In the external validation cohort, deidentified serum samples were collected from each participant recruited from Centre Hospitalier Universitaire Sainte-Justine, Montreal, enrolled in DFCI protocol 16-001; samples were frozen within 1 hour of collection.

### Lipidomics.

Plasma samples from the discovery cohort and serum samples from the validation cohort were submitted to Metabolon for analysis with the Complex Lipid Platform. Lipid extraction was performed as previously described (25).

### Proteomics.

Serum samples from the validation cohort (controls, *n* = 48; cases, *n* = 52) were submitted to Olink for analysis with the Olink 3072 platform for proteomics analysis. The Olink platform utilizes proximity extension assay technology, enabling simultaneous quantification of multiple protein analytes with high sensitivity and specificity (67).

### Statistics.

Initial and postinduction time points and the ratio between the 2 time points for each lipid were log_2_ transformed to better satisfy the normality assumption. No blood samples were excluded from analysis; only lipid species with a missing rate greater than 50% at each time point and in each group were filtered out, resulting in 853 lipid species for the analysis in the discovery cohort. PCA was conducted and visualized by time points using the first 2 principal components. The same PCA analysis was conducted at each time point separately and visualized by case and control groups. Two-sample, 2-tailed *t* tests with unequal variance were performed to compare the lipid concentrations between cases and controls at each time point. For each lipidomic sampling condition, differential abundance analysis was conducted using a linear mixed-effects model to account for lipids measured at 2 time points, adjusting for age, BMI (age-adjusted percentile), and initial risk for ALL to identify lipids that exhibited significant changes between cases and controls and over time. *P* values and *q* values were obtained using the Benjamini-Hochberg method for FDR control. Statistical significance thresholds were prespecified and depended on the analysis (detailed below). In class enrichment analysis, the numbers of significantly (*P* < 0.05) and nonsignificantly different lipids (*P* ≥ 0.05) between cases and controls in a specific lipid class compared with all other lipid classes were analyzed using a 2 × 2 contingency table (1-tailed Fisher’s exact test). We used a 1-tailed test because our hypothesis was that there were more, not less, significantly changed lipids. For each lipid class, a multivariable logistic regression model was performed to examine the association between AAP risk and the lipid class at each time point, adjusting for age, BMI, and initial risk for ALL. The OR with a 95% CI was calculated. WGCNA for lipid species was conducted for cases and controls separately at each time point with the soft threshold setting as 8 at the initial and 14 at the postinduction time point, and minimum module size as 25. Similar modules were merged. The WGCNA module assignments are independent for each analysis, indicating that a specific module identified in one analysis does not correspond to the same set of lipids or features in another analysis. Lipids not clustered into any modules are uncorrelated with any other modules. The proportion of uncorrelated lipids within each lipid group was compared between cases and controls using 2-sided, 2-sample proportion tests at each time point. Module preservation analysis was performed for lipid species at each time point, using the modules identified from the control group as the reference group to compare the correlation patterns in lipids between cases and controls. Nonpreserved modules were identified using median ranks and Z-summary statistics, with lower Z-summary and higher median rank showing less preserved modules. Z-summary less than 2 indicates unpreserved modules, between 2 and 10 indicates moderately preserved modules, and greater than 10 indicates preserved modules. Median ranks were checked along with Z-summary to ensure that the unpreserved modules identified as Z-summary were a function of the module. The module eigengene was calculated for the whole discovery data using the modules identified from the control group. We employed the same statistical analyses for lipidomics in the external validation cohort. For proteomics, we identified differentially expressed proteins using the same statistical methods applied in the lipidomics analysis. For pathway enrichment analysis, we utilized Fisher’s exact test, as detailed in the lipid class enrichment analysis. We examined the associations between LPC lipids and cytokine in the validation cohort. Eight LPC species were selected from differential analysis using a *P* value less than 0.05 after induction. Linear mixed-effects models were performed separately for cases and controls to examine the associations between each lipid and each cytokine, incorporating cytokine-by-time interaction term. The models were adjusted for age, sex, and ALL final risk. The statistical significance of the association between each lipid and each cytokine was tested at each induction time. *q* values were obtained using the Benjamini-Hochberg method within each lipid and within each induction time for FDR control. *q* values less than 0.05 were considered as statistically significant associations between a lipid and a cytokine. The results were visualized using network plots. Pearson’s correlations between lipids were calculated with edges to denote the pairwise significant relationships (*P* < 0.05). The edges between lipids and cytokines denote the significant associations from the linear mixed-effects models. Red and blue dots in cytokines denote the positive or negative associations between lipids and cytokines.

### Study approval of the DFCI ALL protocols.

The DFCI ALL Consortium, established in 1981, recruits children and adolescents (aged 1–21 years) with newly diagnosed ALL into clinical trials. All protocols received approval from the IRBs at DFCI and each participating institution. Written informed consent was obtained from each participant’s parent or legal guardian, as well as patient assent when appropriate, before enrollment. DFCI protocols 05-001, 11-001, and 16-001 are registered at ClinicalTrials.gov with identifiers NCT00400946, NCT01574274, and NCT03020030, respectively.

### Data availability.

All data associated with this study are present in the paper, the supplemental materials, or the Supporting Data Values file (values underlying all graphs and reported means), or are posted publicly (https://www.ebi.ac.uk/metabolights/MTBLS2394 and https://www.ncbi.nlm.nih.gov/geo/query/acc.cgi?acc=GSE181157). Proteomics data have been deposited in the PRIDE (68) repository under the dataset identifier PAD000033. Custom R code used for analysis is available from the corresponding authors upon reasonable request.

## Author contributions

CYT, NB, LW, YD, and SZH conceived and designed the study. CYT, NB, SG, AGJ, YD, and SZH analyzed and interpreted the data. THT, YT, and LBS were involved in clinical trial design and implementation and provided the clinical samples. BL, MAEH, SDM, and EJL contributed essential insights to the study. CYT, NB, THT, GS, YD, and SZH wrote the original manuscript. All authors participated in intellectual discussions and contributed to the final manuscript. CYT and NB contributed equally as co–first authors; co–first author order was assigned based on overall project leadership and primary manuscript development (CYT) and primary responsibility for statistical analysis (NB).

## Conflict of interest

SZH is a consultant for Kiniksa Pharmaceuticals and Triveni Bio.

## Funding support

The following organizations provided financial support:

- Servier Pharmaceuticals (IIT-95014-027-USA to SZH).
- Stanford Diabetes Research Center (P30DK116074 to SZH).
- Stanford’s SPARK Translational Research Program (mentorship to SZH).
- Fonds de Recherche du Québec – Santé (to THT).
- Fondation Charles-Bruneau (to THT).
- Leukemia & Lymphoma Society of Canada (to THT).

## Supplementary Material

Supplemental data

ICMJE disclosure forms

Supporting data values

## Figures and Tables

**Table 1:**
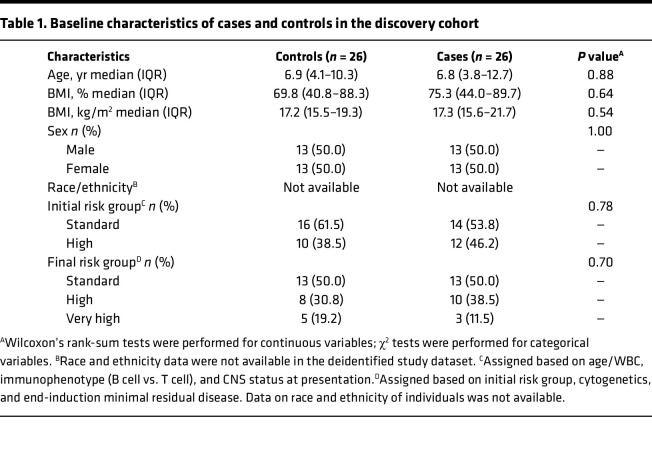
Baseline characteristics of cases and controls in the discovery cohort

**Table 2:**
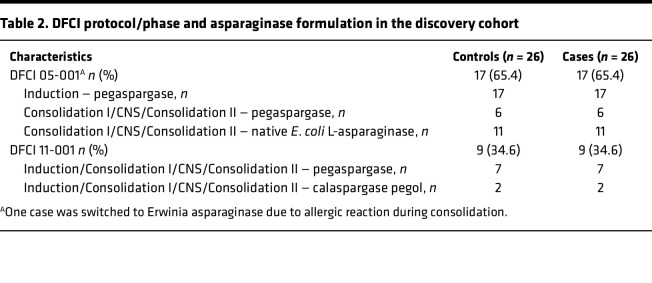
DFCI protocol/phase and asparaginase formulation in the discovery cohort

**Table 3:**
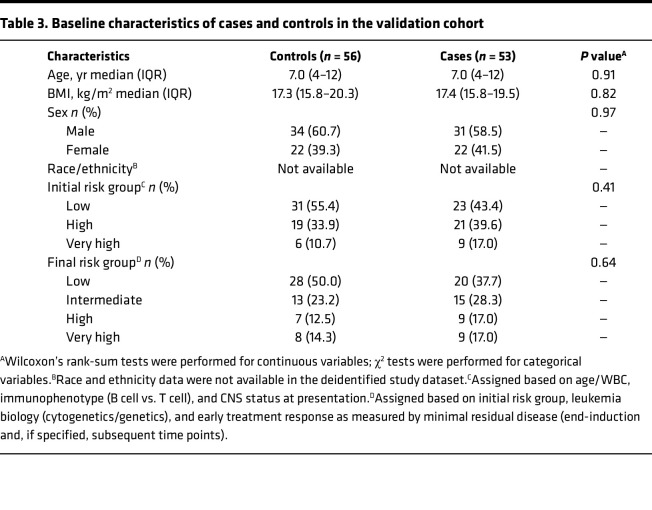
Baseline characteristics of cases and controls in the validation cohort

**Table 4:**
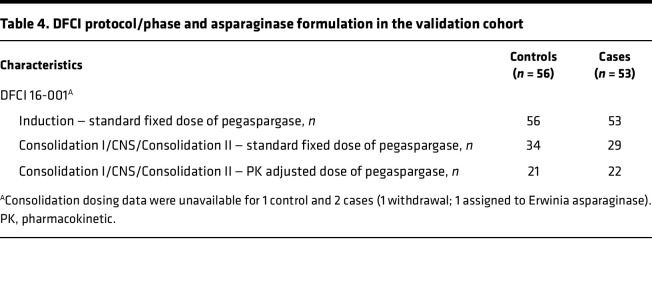
DFCI protocol/phase and asparaginase formulation in the validation cohort

## Version 1 05/01/2026

In-Press Preview

## Version 2 06/22/2026

Electronic publication
