# Supplementary material for: A lipid-immune network signature defines susceptibility to asparaginase-associated pancreatitis
Source: JCI Insight. 2026 Apr 28;11(12):e202662. doi: 10.1172/jci.insight.202662 (PMC13313482; doi:10.1172/jci.insight.202662)
Supplement: Supplemental data [file jciinsight-11-202662-s253.pdf]

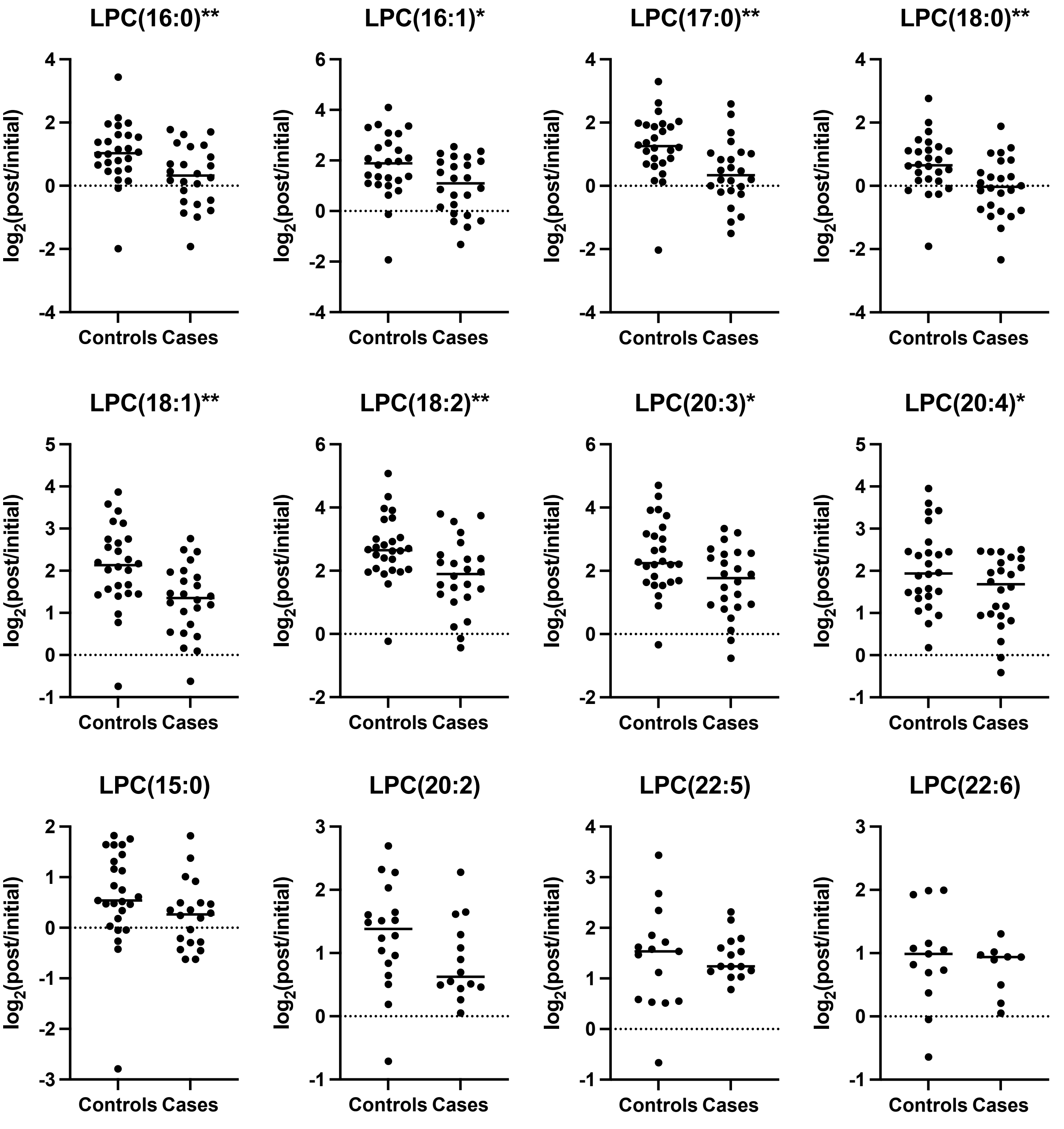

Supplemental Figure 1. Individual LPC species show attenuated induction-associated increases in AAP cases.

Eight LPC species exhibit significantly reduced post-induction to initial ratios in cases compared with controls. *P < 0.05, **P < 0.01; unpaired t test with Welch's correction.

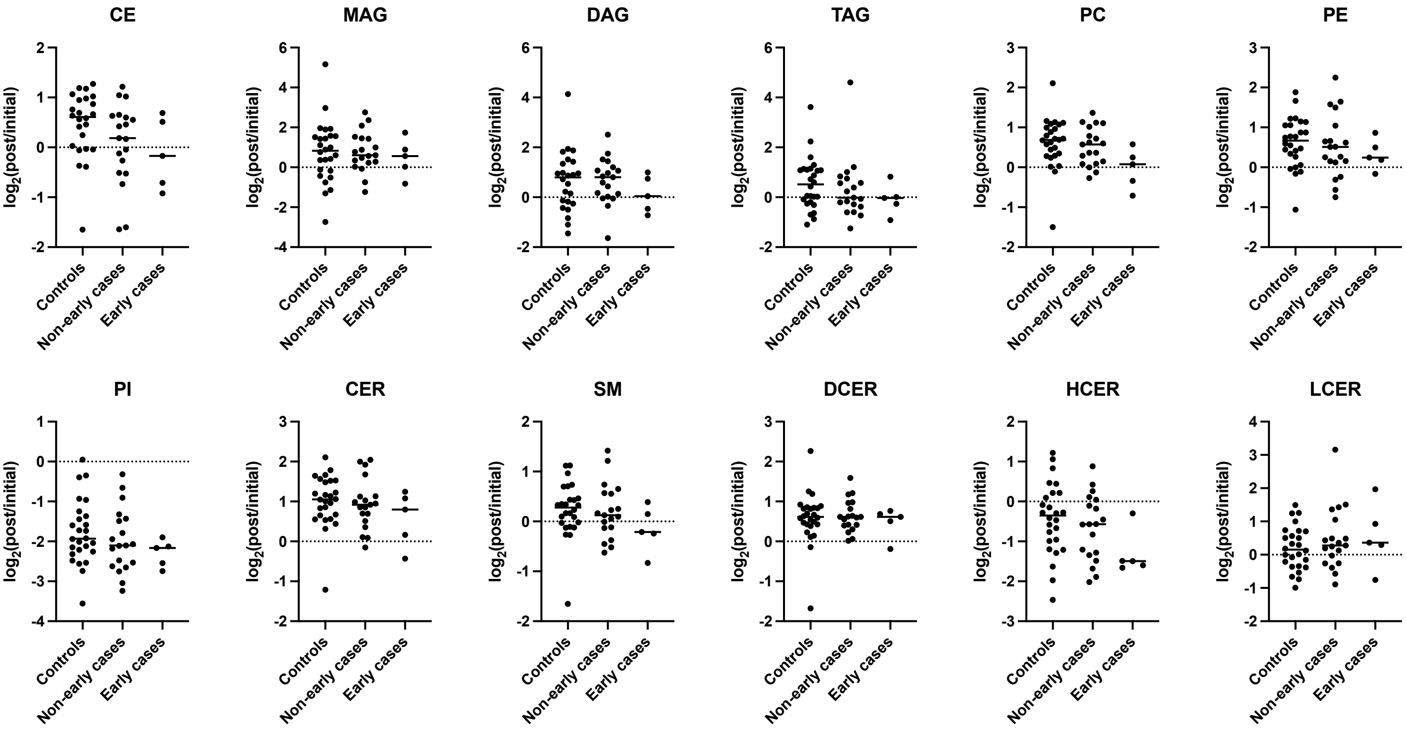

Supplemental Figure 2. Lipid classes other than LPC and LPE do not show timing-dependent differences.

Linear trend testing shows no significant differences across controls, non-early cases, and early cases for lipid classes other than LPC and LPE.

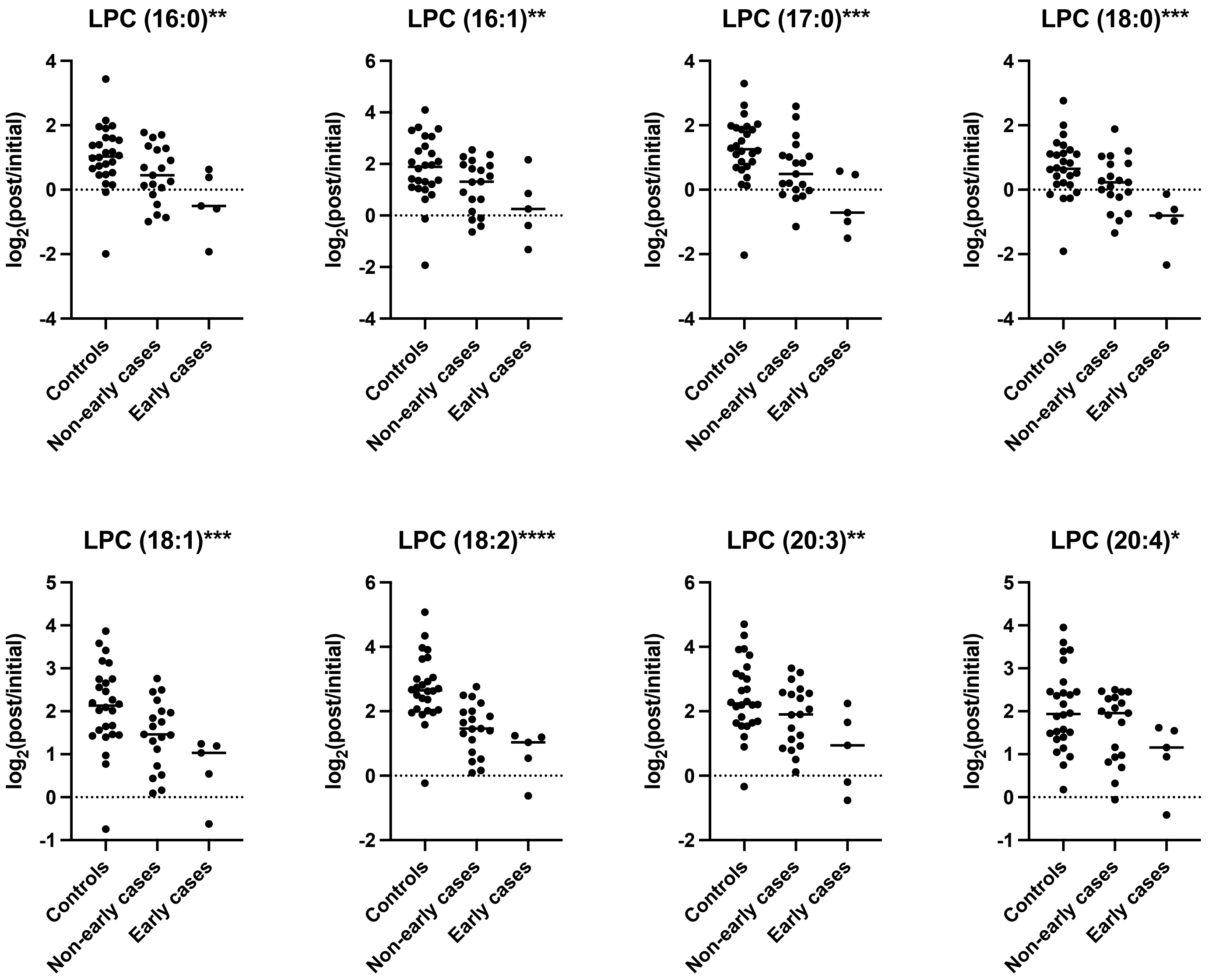

Supplemental Figure 3. Reduced post/initial LPC ratios in cases across eight LPC species, with the lowest ratios in early cases.

Post-induction to initial ratios for eight LPC species, stratified by group (controls, non-early cases, early cases).**P < 0.01, ***P < 0.001, ****P < 0.0001, linear trend test.

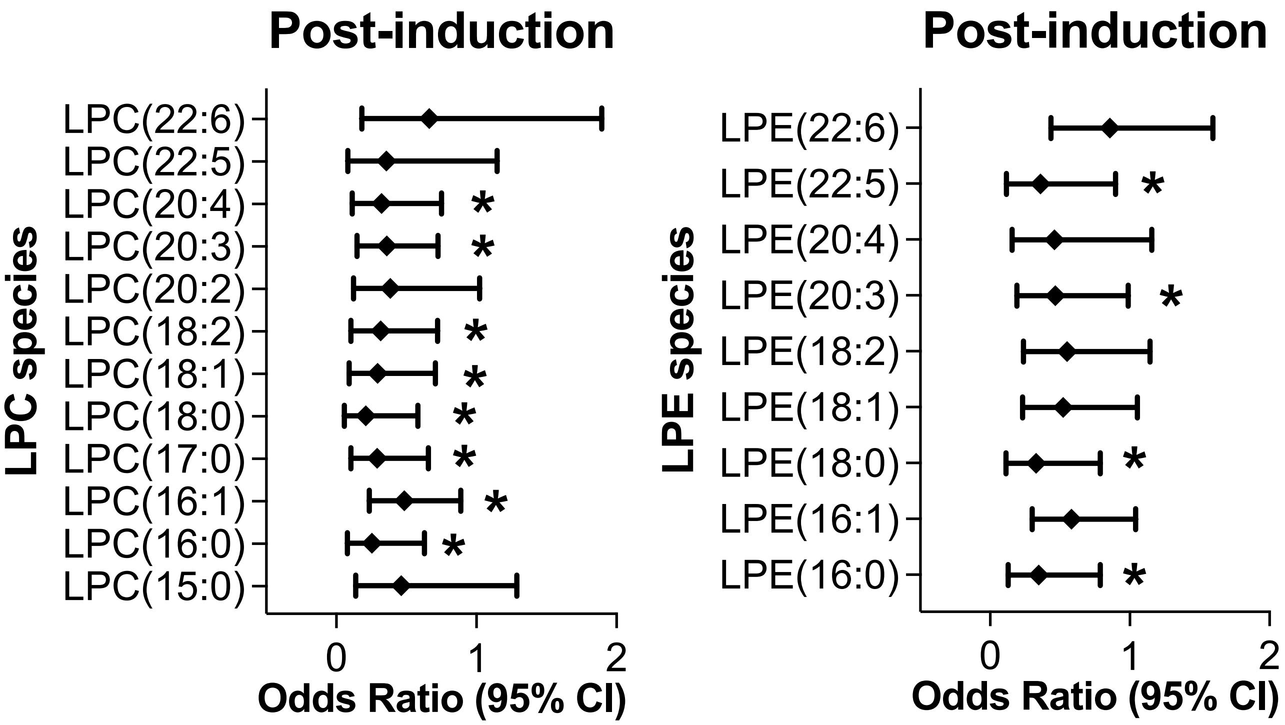

Supplemental Figure 4. Selected post-induction LPC and LPE species are associations with reduced AAP risk.

Forest plots display odds ratios (ORs) and 95% confidence intervals (CIs) for AAP per 1-unit increase in $\log_2$ post-induction lipid concentration for individual LPC (left) and LPE (right) species. *indicates 95% confidence interval (CI) does not cross 1.

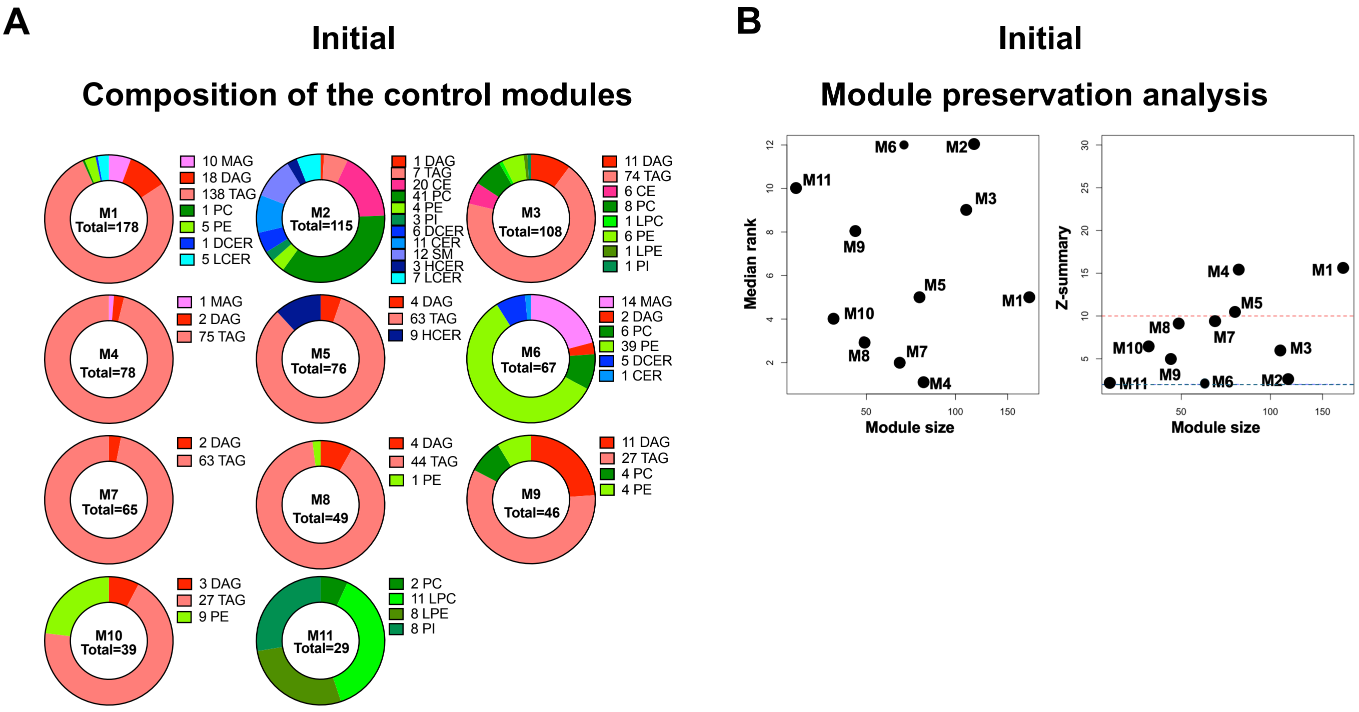

Supplemental Figure 5. WGCNA identifies non-preserved lipid modules at baseline.

(A) Lipid module composition at the initial timepoint in controls.

(B) Module preservation analysis identifies three non-preserved modules in cases, including one (M11) enriched for LPC species.

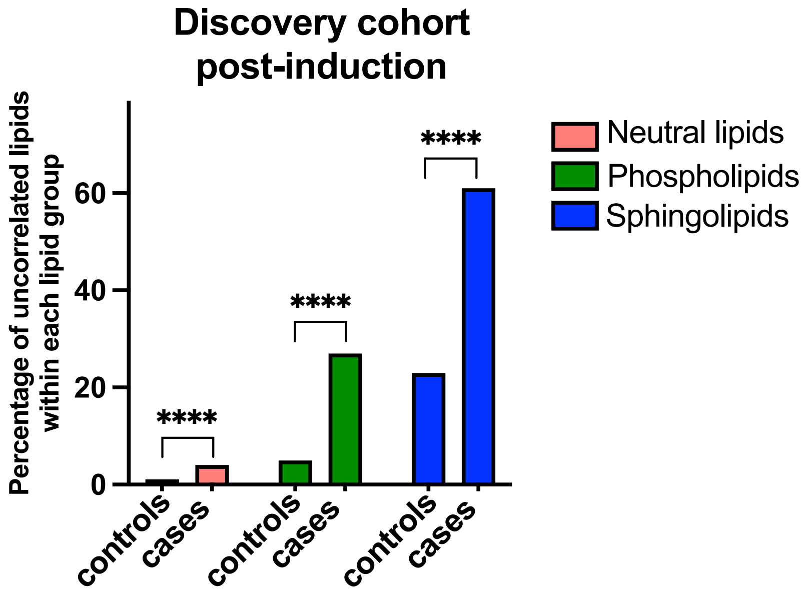

Supplemental Figure 6. Increased proportion of uncorrelated lipid species in AAP cases post-induction in the discovery cohort.

Cases exhibit a significantly higher proportion of uncorrelated lipid species compared with controls, consistent with network-level dysregulation. ****P < 0.0001.

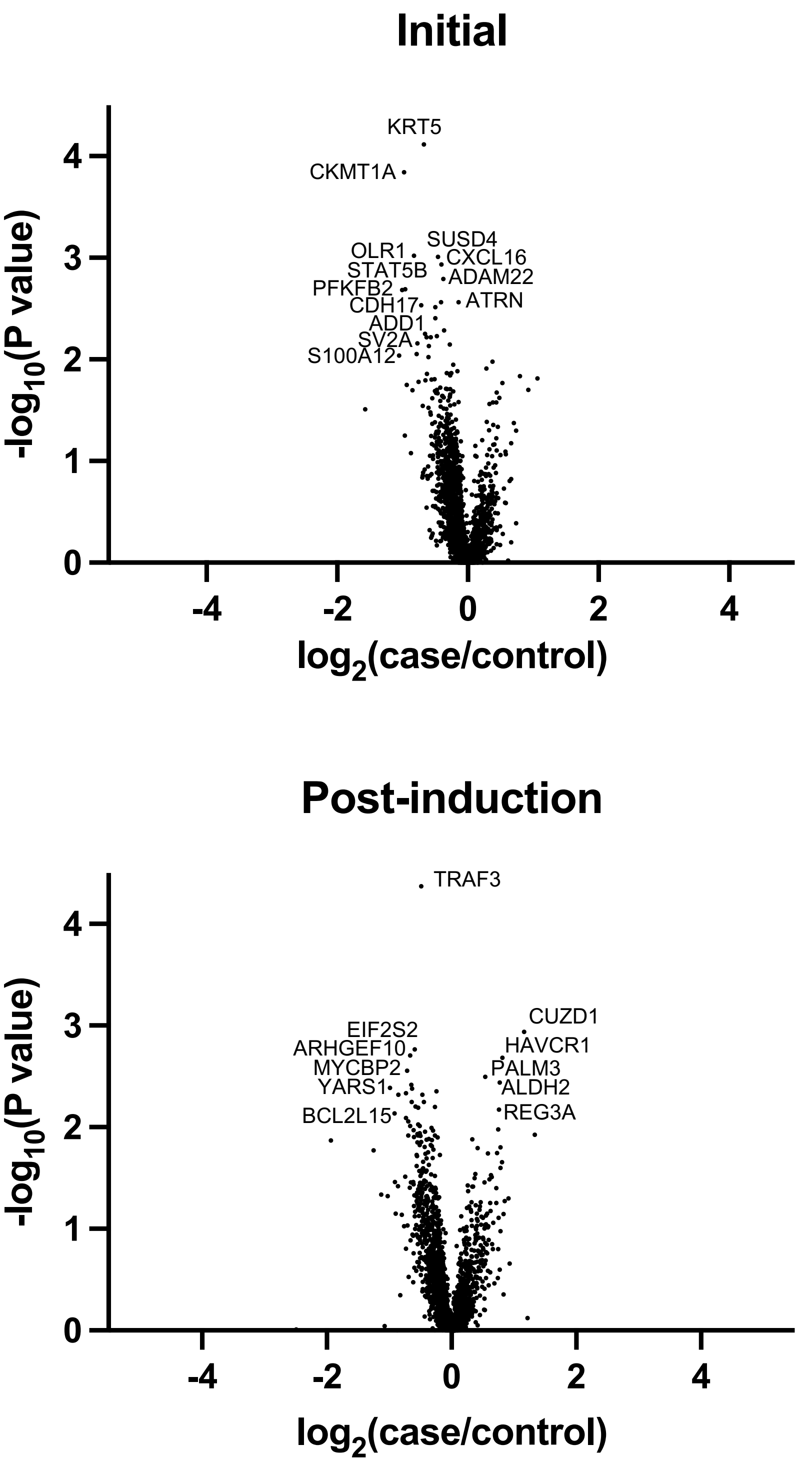

Supplemental Figure 7. Differential protein expression at baseline and post-induction. Volcano plots showing differentially expressed proteins between cases and controls at each timepoint. Only proteins with $P < 0.01$ were annotated where space allowed.

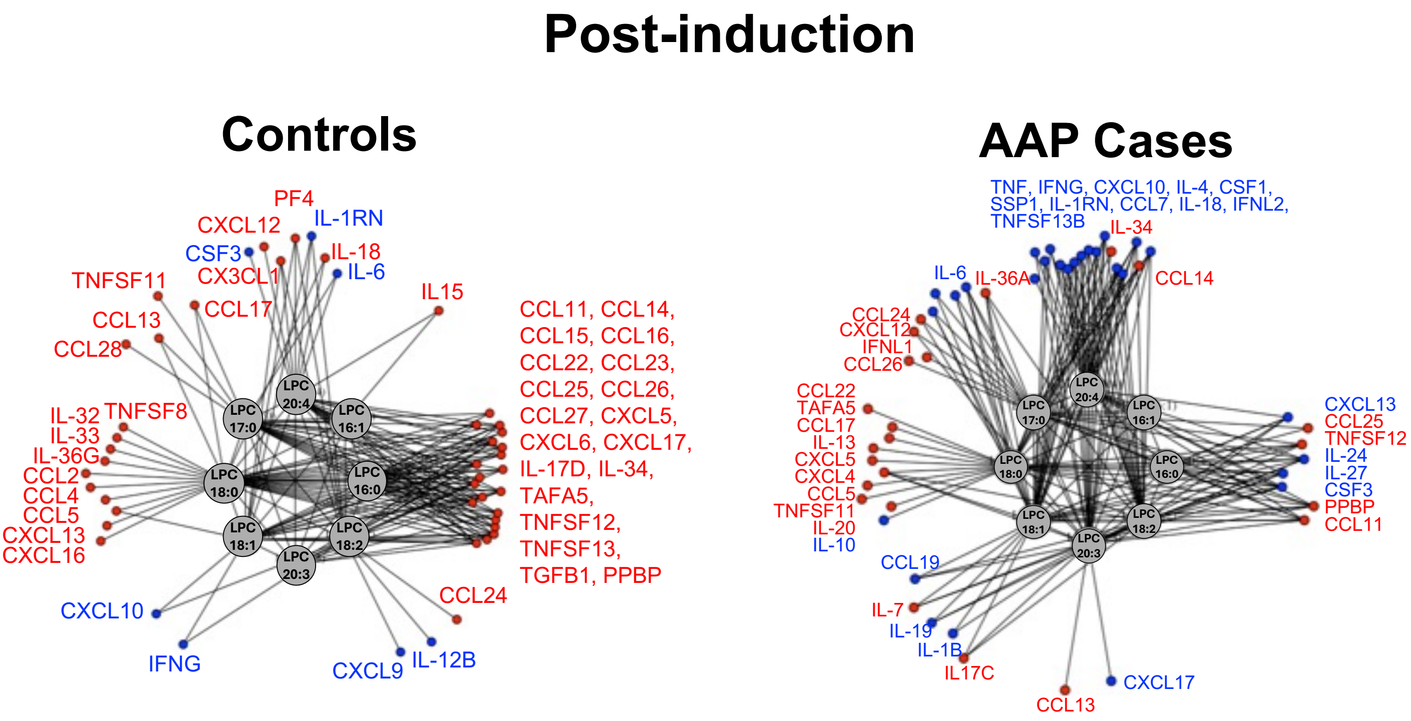

Supplemental Figure 8. Divergent LPC–cytokine association patterns in cases and controls.

Network plots illustrate widespread remodeling of LPC–cytokine associations in cases relative to controls post-induction. Each circle represents a cytokine, with red indicating a significant positive association and blue indicating a negative association between the cytokine and the LPC ($Q < 0.05$).

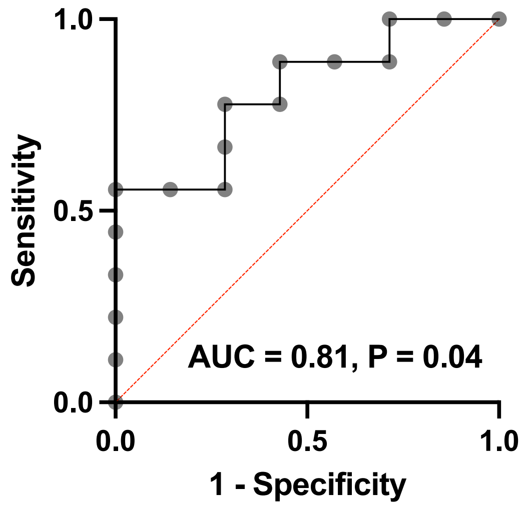

Supplemental Figure 9. IL-18/LPC(18:0) ratio discriminates AAP risk in very high-risk ALL.

Receiver operating characteristic analysis demonstrates discriminatory performance of the IL-18/LPC(18:0) ratio within the very high-risk subgroup.

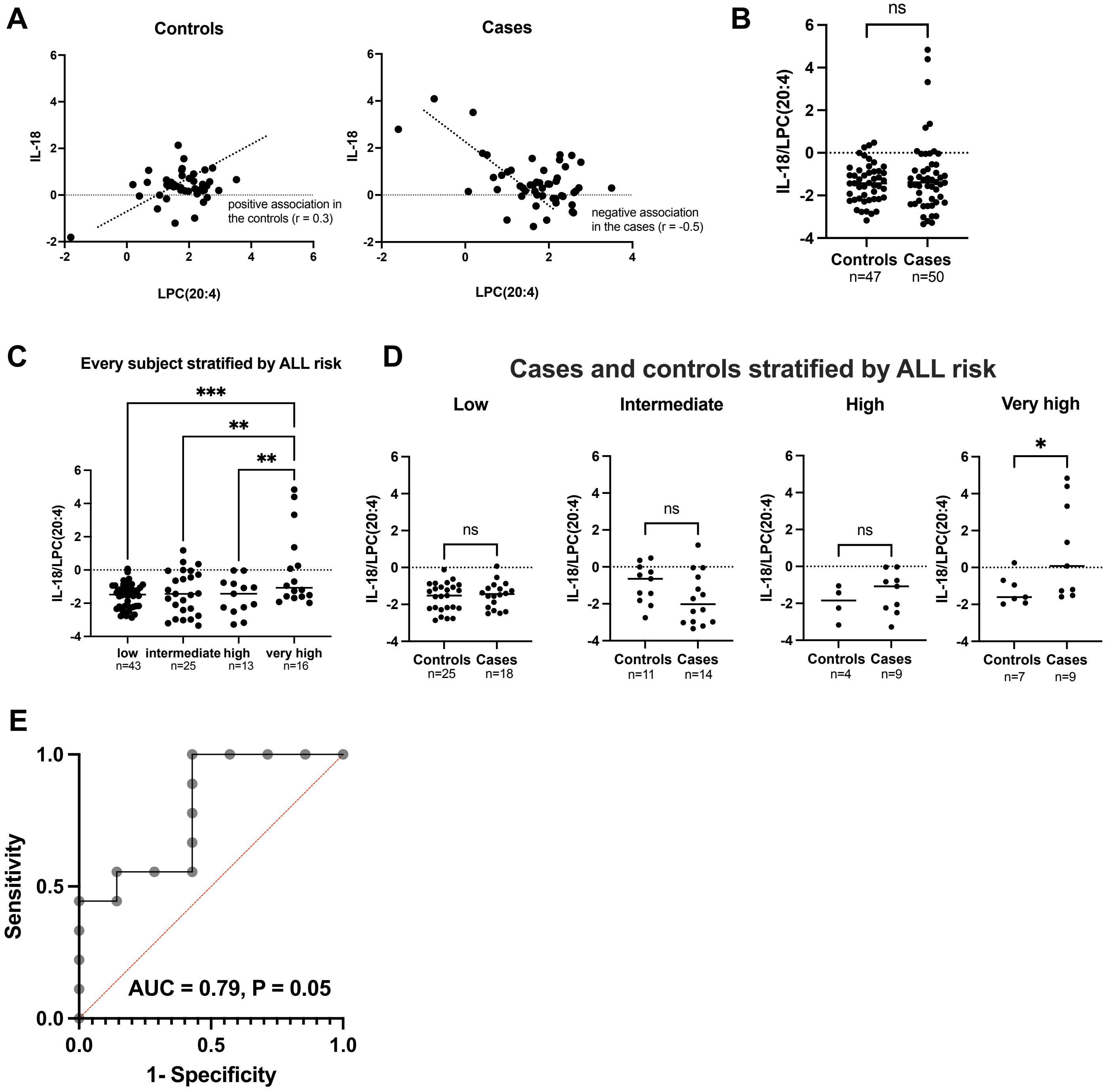

Fig. S10. Parallel IL-18/LPC(20:4) association patterns mirror LPC(18:0) findings.

Correlation (A), ratio (B), and stratified analyses (C-E) show similar IL-18–LPC(20:4) dynamics, reinforcing the IL-18/LPC signature.

Supplemental Table 1. Structural classification of lipids: backbone, head group, and conjugation properties

Lipid group	Lipid class	Backbone	Head group	Number of conjugated fatty acid	Number of conjugated phosphate
neutral lipid	monoacylglycerol (MAG)	glycerol	NA	1	0
	diacylglycerol (DAG)	glycerol	NA	2	0
	triacylglycerol (TAG)	glycerol	NA	3	0
	cholesteryl ester (CE)	sterol	NA	1	0
phospholipid	phosphatidylcholine (PC)	glycerol	choline	2	1
	lysophosphatidylcholine (LPC)	glycerol	choline	1	1
	phosphatidylethanolamine (PE)	glycerol	ethanolamine	2	1
	lysophosphatidylethanolamine (LPE)	glycerol	ethanolamine	1	1
	phosphatidylinositol (PI)	glycerol	inositol	2	1
sphingolipid	dihydroceramide (DCER)	sphinganine	NA	1	0
	ceramide (CER)	sphingosine	NA	1	0
	sphingomyelin (SM)	sphingosine	phosphocholine	1	1
	hexosylceramide (HCER)	sphingosine	hexose	1	0
	lactosylceramide (LCER)	sphingosine	lactose	1	0

Supplemental Table 2. Overview of biostatistical methods for data analysis

Methods	Purposes	Pros	Cons	Figure	Table
Principal Component Analysis (PCA)	Exploratory analysis and visualization of lipids on a two-dimensional plot by timepoints and subject groups, using the first two principal components of lipids that explain majority of variation in all lipids.	Intuitive by plotting the lipids on a two-dimensional plot through dimension deduction for identifying group and time differences, batch effects and outliers; useful for initial examination.	Subjective way to examine the differences between subjects groups or timepoints; provides limited interpretability.	1E, G, 3D	
Linear mixed-effects regression model	Rigorous statistical modeling approach to test lipid expression differences between the subject groups and over time by taking lipids measured at multiple timepoints into account, adjusting for baseline covariates.	Accounts for time, group, and interaction effects in one model.	Power may be reduced with small smaple sizes, especially in the discovery cohort	2A, 4A	S3-4, 9
Class enrichment analysis through one-sided Fisher exact tests	To examine whether the lipid class containing differentially represented lipids are statistically significant enriched.	A rigorous way to examine whether the lipid class contains differentially represented lipids is beyond what would be expected by chance; one-sided tests answer the hypothesis whether the lipid class is enriched with significantly differentially represented lipids ("over representation") compared to two-sided tests which only answer if there is any differences in the number of significant differentially represented lipids.	Require prior hypothesis on the direction of the test when using the one-sided testing approach; lack identification of under respresentation.	2B	
Multivariable logistic regression	To examine the effect of lipids on the risk of developing pancreatitis, adjusting for baseline covariates.	A rigorous parametric regression to examine the association between the covariate and a binary outcome; odds ratio with 95% confidence interval can be obtained with intuitive interpretation for developing pancreatitis.	Require a moderate sample size especially for rare events.	2E	

WGCNA (weighted gene co-expression networ k analysis)	Identify lipid clusters and assess the correlation patterns between subject groups.	It has a rigorous rationale and statistical procedure through weighted correlation and topology to identify the correlation patterns and give information about "whether correlations within lipids are different between two groups". Module eigengene can be further used as a representative of a module to perform more analysis on module level. More rigorous than Spearman rank correlation which can only subjectly see the differences in correlation patterns between groups.	Sensitive to the choice of some parameters (e.g., soft threshold, monimum node size); subjective in determining the unpreserved module as Z-summary is sensitive to the module size and the threshold is subjective, but still more objective than visually comparing Spearman correlations between groups.	3E-F, S5	S5-6, 8
Two-sided two-sample proportion test	Examine the proportion of lipids uncorrelated with any other modules within each lipid group between cases and controls.	easy interpretation of comparing two proportions in two independent groups; widely used.	Has size requirements especially requiring larger sample size if the proportion is close to 0 or 1.	S6	
Pathway enrichment analysis through one-sided Fisher exact tests	To examine whether the pathway containing differentially expressed proteins are statistically significant enriched.	A rigorous way to examine whether the pathway containing differentially expressed proteins is beyond what would be expected by chance; one-sided tests answer the hypothesis whether the pathway is enriched with significant differentially expressed proteins("over representation") compared to two-sided tests which only answer if there is any differences in the number of significant differentially expressed proteins.	Require prior hypothesis on the direction of the test when using the one-sided testing approach; lack identification of under respresentation.	4B	

Supplemental Table 3. Linear mixed-effects regression analysis of lipid class concentrations at initial and post-induction timepoints, and their ratios (post/initial) between cases and controls

Lipid group	Lipid class	Initial			Post-induction			Post/intial ratio		
		tstat	P value	Q value	tstat	P value	Q value	tstat	P value	Q value
neutral lipid	DAG	0.814	0.661	0.993	-0.459	0.876	0.876	-0.056	0.955	0.970
	MAG	-0.108	0.993	0.993	-0.476	0.867	0.876	0.038	0.970	0.970
	TAG	-0.287	0.949	0.993	-0.666	0.757	0.876	-0.868	0.390	0.682
	CE	0.609	0.792	0.993	-1.951	0.105	0.421	-2.105	0.041	0.191
phospholipid	PC	0.505	0.851	0.988	-1.757	0.16	0.26	-1.045	0.302	0.604
	**LPC**	0.696	0.738	0.988	-3.584	**0.001**	**0.005**	-2.850	**0.007**	**0.092**
	PE	-0.140	0.988	0.988	-1.363	0.321	0.402	-0.544	0.589	0.750
	LPE	-0.412	0.899	0.988	-2.384	**0.038**	**0.095**	-2.268	0.028	0.191
	PI	0.670	0.755	0.988	-1.195	0.415	0.42	-1.109	0.273	0.604
sphingolipid	DCER	-0.729	0.717	0.950	-0.974	0.554	0.554	0.290	0.773	0.902
	CER	-1.106	0.469	0.950	-1.863	0.127	0.318	-0.717	0.477	0.743
	SM	0.657	0.763	0.950	-1.301	0.354	0.443	-0.587	0.560	0.750
	HCER	0.360	0.922	0.950	-1.493	0.258	0.431	-1.345	0.185	0.604
	LCER	-0.284	0.950	0.950	2.127	0.071	0.318	1.236	0.223	0.604

tstat: the ratio of the difference in a number’s estimated value from its assumed value to its standard error.

Supplemental Table 4. Differentially presented lipids species identified post-induction (P < 0.05)

Lipid group	Lipid species	log_2(case/control)	-log(P value)
neutral lipid	DAG(14:0/20:0)	-0.77	1.60
	TAG54:5-FA18:0	-0.70	1.44
	TAG54:6-FA18:2	-0.72	1.43
	TAG54:7-FA18:1	-0.69	1.48
	TAG54:7-FA18:2	-0.84	1.62
	TAG54:7-FA18:3	-0.81	1.61
	TAG54:8-FA18:2	-0.95	1.97
	TAG54:8-FA18:3	-0.99	1.85
	TAG55:6-FA20:3	0.11	1.50
	CE(12:0)	-0.94	1.68
	CE(14:0)	-0.52	1.44
	CE(15:0)	-0.54	1.92
	CE(17:0)	-0.56	1.81
phospholipid	PC(14:0/20:3)	-0.69	2.54
	PC(14:0/20:4)	-0.40	1.55
	PC(16:0/20:2)	-0.44	1.84
	PC(16:0/20:3)	-0.53	1.76
	PC(16:0/20:5)	-0.85	1.36
	PC(16:0/22:4)	-0.41	1.45
	PC(16:0/22:5)	-0.47	1.61
	PC(18:0/20:3)	-0.47	1.52
	PC(18:0/22:5)	-0.47	1.86
	PC(18:2/20:3)	-0.40	1.94
	PC(18:2/20:4)	-0.32	1.69
	LPC(16:0)	-0.59	2.89
	LPC(16:1)	-0.62	1.98
	LPC(17:0)	-0.66	2.65
	LPC(18:0)	-0.59	2.67
	LPC(18:1)	-0.57	2.32
	LPC(18:2)	-0.54	1.90
	LPC(20:3)	-0.71	2.62
	LPC(20:4)	-0.56	2.07
	PE(18:2/16:1)	-0.52	1.99
	PE(O-18:0/20:3)	-0.54	1.82
	PE(P-16:0/18:2)	-0.64	2.23
	PE(P-16:0/20:3)	-0.76	2.68
	PE(P-18:0/18:2)	-0.65	2.32
	PE(P-18:0/20:3)	-0.85	2.67
	PE(P-18:0/20:5)	-0.93	2.00
	PE(P-18:1/18:2)	-0.54	1.71
	PE(P-18:1/20:3)	-0.75	2.18
	LPE(16:0)	-0.50	2.22
	LPE(18:0)	-0.51	2.04
	LPE(22:5)	-0.60	1.34
	PI(18:0/20:3)	-0.39	1.48
sphingolipid	CER(24:0)	-0.42	1.63
	DCER(22:2)	-0.70	2.04
	LCER(24:1)	1.10	1.62

In the Metabolon lipid nomenclature system, the letter indicates the lipid class, while the content in parentheses specifies the conjugated fatty acid. The first number represents the number of carbon atoms in the fatty acid chain, and the second number denotes the number of double bonds.

Supplemental Table 5. Non-preserved lipid species identified at initial in the discovery cohort

Lipid group	Lipid Species
neutral lipid	MAG(14:0)
	MAG(16:0)
	MAG(17:0)
	MAG(18:0)
	MAG(18:1)
	MAG(18:2)
	MAG(18:3)
	MAG(20:1)
	MAG(20:2)
	MAG(20:3)
	MAG(20:4)
	MAG(22:4)
	MAG(22:5)
	MAG(22:6)
	DAG(16:1/20:0)
	DAG(16:1/20:2)
	DAG(16:1/22:6)
	TAG55:7-FA15:0
	TAG58:6-FA16:0
	TAG58:7-FA20:4
	TAG58:8-FA20:3
	TAG58:8-FA20:4
	TAG58:9-FA20:4
	TAG58:10-FA20:4
	CE(12:0)
	CE(14:0)
	CE(14:1)
	CE(15:0)
	CE(16:0)
	CE(16:1)
	CE(17:0)
	CE(18:0)
	CE(18:1)
	CE(18:2)
	CE(18:3)
	CE(18:4)
	CE(20:2)
	CE(20:3)
	CE(20:4)
	CE(22:0)
	CE(22:1)
	CE(22:4)
	CE(22:5)
	CE(24:1)
	PC(14:0/18:1)
	PC(14:0/18:2)
	PC(14:0/20:3)
	PC(14:0/20:4)
	PC(15:0/18:1)
	PC(15:0/18:2)
	PC(16:0/16:0)
	PC(16:0/16:1)

	PC(16:0/18:0)
	PC(16:0/18:1)
	PC(16:0/18:2)
	PC(16:0/20:1)
	PC(16:0/20:2)
	PC(16:0/20:3)
	PC(16:0/20:4)
	PC(16:0/22:4)
	PC(16:0/22:5)
	PC(17:0/18:1)
	PC(17:0/18:2)
	PC(17:0/20:3)
	PC(17:0/20:4)
	PC(18:0/16:1)
	PC(18:0/18:0)
	PC(18:0/18:1)
	PC(18:0/18:2)
	PC(18:0/20:2)
	PC(18:0/20:3)
	PC(18:0/20:4)
	PC(18:0/22:4)
	PC(18:0/22:5)
	PC(18:1/16:1)
	PC(18:1/18:1)
	PC(18:1/18:2)
	PC(18:1/20:2)
	PC(18:1/20:3)
	PC(18:1/20:4)
	PC(18:1/22:4)
	PC(18:1/22:5)
	PC(18:2/16:1)
	PC(18:2/18:2)
	PC(18:2/18:3)
	PC(18:2/20:2)
	PC(18:2/20:3)
	PC(18:2/20:4)
	PC(18:2/22:5)
	PC(20:0/18:1)
	PC(20:0/18:2)
	PC(20:0/20:3)
	PC(20:0/20:4)
	LPC(15:0)
	LPC(16:0)
	LPC(16:1)
	LPC(17:0)
	LPC(18:0)
	LPC(18:1)
	LPC(18:2)
	LPC(20:2)
	LPC(20:3)
	LPC(20:4)
	LPC(22:5)
phospholipid	PE(18:0/18:0)
	PE(18:0/18:1)
	PE(18:0/20:2)
	PE(18:1/16:1)

PE(18:1/20:4)
PE(18:1/22:5)
PE(18:2/16:1)
PE(O-16:0/18:1)
PE(O-16:0/18:2)
PE(O-16:0/20:4)
PE(O-16:0/22:4)
PE(O-16:0/22:5)
PE(O-16:0/22:6)
PE(O-18:0/18:1)
PE(O-18:0/18:2)
PE(O-18:0/20:3)
PE(O-18:0/20:4)
PE(O-18:0/22:4)
PE(O-18:0/22:5)
PE(O-18:0/22:6)
PE(P-16:0/18:1)
PE(P-16:0/18:2)
PE(P-16:0/20:3)
PE(P-16:0/20:4)
PE(P-16:0/22:4)
PE(P-16:0/22:5)
PE(P-16:0/22:6)
PE(P-18:0/16:0)
PE(P-18:0/18:1)
PE(P-18:0/18:2)
PE(P-18:0/20:3)
PE(P-18:0/20:4)
PE(P-18:0/22:5)
PE(P-18:0/22:6)
PE(P-18:1/16:0)
PE(P-18:1/18:1)
PE(P-18:1/18:2)
PE(P-18:1/20:3)
PE(P-18:1/20:4)
PE(P-18:1/22:4)
PE(P-18:1/22:5)
PE(P-18:1/22:6)
PE(P-18:2/20:4)
LPE(16:0)
LPE(16:1)
LPE(18:0)
LPE(18:1)
LPE(18:2)
LPE(20:3)
LPE(20:4)
LPE(22:5)
PI(16:0/18:1)
PI(16:0/18:2)
PI(16:0/20:4)
PI(18:0/16:1)
PI(18:0/18:1)
PI(18:0/18:2)
PI(18:0/20:3)
PI(18:0/20:4)
PI(18:1/18:1)

	PI(18:1/18:2)
	PI(18:1/20:4)
sphingolipid	DCER(16:0)
	DCER(18:0)
	DCER(18:1)
	DCER(20:0)
	DCER(20:1)
	DCER(22:0)
	DCER(22:1)
	DCER(22:2)
	DCER(24:0)
	DCER(24:1)
	DCER(26:1)
	CER(14:0)
	CER(16:0)
	CER(18:0)
	CER(18:1)
	CER(20:0)
	CER(20:1)
	CER(22:0)
	CER(22:1)
	CER(24:0)
	CER(24:1)
	CER(26:0)
	CER(26:1)
	SM(14:0)
	SM(16:0)
	SM(18:0)
	SM(18:1)
	SM(20:0)
	SM(20:1)
	SM(22:0)
	SM(22:1)
	SM(24:0)
	SM(24:1)
	SM(26:0)
	SM(26:1)
	HCER(18:1)
	HCER(20:1)
	HCER(22:1)
	LCER(14:0)
	LCER(16:0)
	LCER(18:1)
	LCER(20:1)
	LCER(24:0)
	LCER(24:1)
	LCER(26:0)

Supplemental Table 6. Uncorrelated lipid species identified post-induction in the discovery cohort

Lipid group	Controls	Cases
		MAG(14:0)
		MAG(14:1)
		MAG(16:1)
		MAG(18:1)
		MAG(20:2)
		MAG(20:3)
		MAG(20:4)
		MAG(20:5)
		MAG(22:4)
		MAG(22:5)
		MAG(24:1)
	TAG57:9-FA22:6	
		CE(16:0)
		CE(18:0)
neutral lipid		CE(18:1)
		CE(18:3)
	CE(20:0)	CE(20:0)
	CE(20:1)	CE(20:1)
		CE(20:2)
		CE(20:3)
		CE(20:4)
		CE(22:0)
		CE(22:1)
	CE(22:2)	CE(22:2)
		CE(22:4)
		CE(22:5)
		CE(22:6)
		CE(24:0)
		CE(24:1)
		PC(16:0/18:0)
		PC(16:0/20:3)
		PC(16:0/22:5)
		PC(16:0/22:6)
		PC(17:0/18:2)
		PC(17:0/20:3)
		PC(17:0/20:4)
		PC(18:0/18:0)
		PC(18:0/20:3)
		PC(18:0/20:4)
		PC(18:0/22:4)
		PC(18:0/22:5)
		PC(18:0/22:6)
		PC(18:1/20:3)
		PC(18:1/22:5)
		PC(18:2/20:5)
		PC(18:2/22:5)
		PC(18:2/22:6)
		PC(20:0/20:3)
		PC(20:0/20:4)
		LPC(16:0)
		LPC(16:1)
phospholipid	LPC(17:0)	LPC(17:0)
		LPC(18:0)

		LPC(18:1)
	LPC(18:2)	LPC(18:2)
		LPC(20:2)
		LPC(20:3)
		LPC(20:4)
		LPC(22:5)
		LPC(22:6)
		PE(18:1/22:0)
		PE(O-16:0/22:6)
		PE(O-18:0/22:6)
	PE(P-16:0/22:6)	PE(P-16:0/22:6)
	PE(P-18:0/20:5)	
	PE(P-18:0/22:6)	PE(P-18:0/22:6)
	PE(P-18:1/22:6)	PE(P-18:1/22:6)
	LPE(18:2)	LPE(18:2)
		LPE(20:3)
		LPE(20:4)
		LPE(22:5)
		LPE(22:6)
		PI(18:0/18:2)
		PI(18:0/20:3)
	PI(18:0/20:4)	PI(18:0/20:4)
		DCER(18:0)
		DCER(18:1)
		DCER(20:0)
		DCER(20:1)
		DCER(22:0)
		DCER(22:2)
		DCER(24:0)
		DCER(24:1)
	DCER(26:0)	DCER(26:0)
		CER(18:0)
		CER(18:1)
		CER(20:0)
		CER(20:1)
		CER(22:0)
		CER(22:1)
		CER(24:0)
		SM(16:0)
	SM(18:0)	SM(18:0)
	SM(18:1)	SM(18:1)
		SM(20:0)
	SM(20:1)	SM(20:1)
sphingolipid		SM(22:0)
	SM(22:1)	SM(22:1)
	SM(24:0)	SM(24:0)
		SM(24:1)
	SM(26:0)	
	SM(26:1)	
		HCER(14:0)
		HCER(16:0)
		HCER(18:0)
		HCER(18:1)
		HCER(20:0)
		HCER(20:1)
		HCER(22:0)

		HCER(22:1)
		HCER(24:0)
		HCER(24:1)
	LCER(16:0)	LCER(16:0)
	LCER(18:1)	
	LCER(22:0)	
	LCER(24:0)	
	LCER(24:1)	LCER(24:1)
	LCER(26:1)	

Supplemental Table 7. 814 lipid species overlap between discovery and validation cohorts

Lipid group (# species, %)	Lipid class (# species, %)
neutral lipids (627, 77.0)	MAG (25, 3.1)
	DAG (58, 7.1)
	TAG (518, 63.6)
	CE (26, 3.2)
phospholipids (128, 15.7)	PC (59, 7.2)
	LPC (11, 1.4)
	PE (48, 5.9)
	LPE (8, 1.0)
	PI (2, 0.2)
sphingolipids (59, 7.3)	DCER (13, 1.6)
	CER (12, 1.5)
	SM (12, 1.5)
	HCER (12, 1.5)
	LCER, (10, 1.2)

Supplemental Table 8. Non-preserved lipid species identified at post-induction in the validation cohort

neutral lipid	MAG(18:4)
	CE(12:0)
	CE(14:0)
	CE(14:1)
	CE(15:0)
	CE(16:0)
	CE(16:1)
	CE(17:0)
	CE(18:0)
	CE(18:1)
	CE(18:2)
	CE(18:3)
	CE(18:4)
	CE(20:2)
	CE(20:3)
	CE(20:4)
	CE(20:5)
	CE(22:0)
	CE(22:4)
	CE(22:5)
	PC(14:0/18:1)
	PC(14:0/18:2)
	PC(14:0/20:3)
	PC(14:0/20:4)
	PC(15:0/18:1)
	PC(15:0/18:2)
	PC(16:0/18:2)
	PC(16:0/20:1)
	PC(16:0/20:4)
	PC(16:0/20:5)
	PC(16:0/22:4)
	PC(16:0/22:5)
	PC(17:0/18:1)
	PC(17:0/18:2)
	PC(17:0/20:3)
	PC(17:0/20:4)
	PC(18:0/18:2)
	PC(18:0/20:4)
	PC(18:0/20:5)
	PC(18:0/22:4)
	PC(18:0/22:5)
	PC(18:1/18:2)
	PC(18:1/18:3)
	PC(18:1/20:4)
	PC(18:1/20:5)
	PC(18:1/22:4)
	PC(18:1/22:5)
	PC(18:2/16:1)
	PC(18:2/18:2)
	PC(18:2/18:3)
	PC(18:2/20:3)
	PC(18:2/20:4)

phospholipid	PC(20:0/18:2)
	PC(20:0/20:4)
	LPC(15:0)
	LPC(16:0)
	LPC(16:1)
	LPC(17:0)
	LPC(18:0)
	LPC(18:1)
	LPC(18:2)
	LPC(20:2)
	LPC(20:3)
	LPC(20:4)
	LPC(22:5)
	PE(O-16:0/22:5)
	PE(O-18:0/20:4)
	PE(O-18:0/22:5)
	PE(P-16:0/18:1)
	PE(P-16:0/18:2)
	PE(P-16:0/20:3)
	PE(P-16:0/20:4)
	PE(P-16:0/22:4)
	PE(P-16:0/22:5)
	PE(P-18:0/18:1)
	PE(P-18:0/18:2)
	PE(P-18:0/20:3)
	PE(P-18:0/20:4)
	PE(P-18:0/22:4)
	PE(P-18:0/22:5)
	PE(P-18:1/18:1)
	PE(P-18:1/18:2)
	PE(P-18:1/20:3)
	PE(P-18:1/20:4)
	PE(P-18:1/22:5)
	LPE(16:0)
	LPE(18:0)
	LPE(18:1)
	LPE(22:5)
	PI(18:0/20:4)
sphingolipid	DCER(24:0)
	CER(24:0)
	CER(26:0)
	SM(14:0)
	SM(16:0)
	SM(20:0)
	SM(20:1)
	SM(22:0)
	SM(22:1)
	SM(24:0)
	SM(26:0)
	SM(26:1)
	HCER(14:0)
	HCER(16:0)
	HCER(18:0)
	HCER(20:0)
	HCER(22:0)
	HCER(24:0)

HCER(24:1)	;;
LCER(18:1)	
LCER(20:1)	

Lipid species labeled in black were within the non-preserved module (M2), while those labeled in blue were within the modestly non-preserved module (M6).

Supplemental Table 9. Differentially expressed proteins (P < 0.05) in cases and controls across three sampling conditions

Initial	Post-induction	Post/Initial
ACYP1	AARSD1	ACE2
ADAM22	ACAN	ADAMTS4
ADAMTS1	ACE2	ADAMTSL2
ADD1	ACYP1	APCS
ANXA1	AK1	ARHGEF10
APCS	ALDH2	ARTN
APOA1	ALDH5A1	ATP1B3
APOB	AMPD3	BRK1
APOD	ANXA1	C1QTNF1
ARHGEF1	ANXA11	C1QTNF5
ASRGL1	ANXA3	C1S
ATP1B3	APRT	CABP2
AZU1	ARHGEF1	CBS
BRAP	ARHGEF10	CCL19
BRDT	ARHGEF12	CCN4
BRK1	ATXN3	CCN5
CDH17	BCL2L15	CDH4
CEACAM21	BLVRB	CEACAM5
CFB	CA3	CFB
CKMT1A	CALCOCO1	CFHR5
CKMT1B	CC2D1A	CKMT1A
CLU	CCL20	CKMT1B
CNST	CCS	COL18A1
CSPG4	CCT5	COL3A1
CST6	CD300LG	COL4A1
CTHRC1	CDH17	CPXM2
CTSD	CEACAM5	CRX
CXCL16	CEP85	CSF3
DKK3	CHAC2	CSRP3
DLK1	CHMP1A	CUZD1
DNAJB2	CRADD	CXADR
DPP7	CRYGD	CXCL17
DSG3	CSF2	CXCL8_Cardiometabolic
DTX3	CSF2RB	CXCL8_Inflammation
EFNB2	CTSE	CXCL8_Neurology
EIF2AK2	CUZD1	CXCL8_Oncology
ENO2	CXCL13	DUOX2
EPHB6	CXCL8_Inflammation	ECHS1
F13B	CXCL8_Oncology	EDN1
FGF19	DARS1	EFNB2
FGFBP1	DCTN1	EIF5
GBP1	DGKA	FOXO1

GFRA3	DNAJB1	GCLM
GPHA2	DNAJB2	HLA-E
HSPG2	DPP7	ICAM2
HYAL1	DSG3	IFNAR1
ICAM2	DTYMK	IGFL4
ICAM5	ECHS1	IGSF3
IFNAR1	EIF2AK2	IL-18R1
IL-17D	EIF2S2	IL-32
IPCEF1	EIF4E	IL-6_Cardiometabolic
KIRREL2	ENOX2	IL-6_Inflammation
KRT5	ENPP5	IL-6_Neurology
LCN15	ENPP6	IL-6_Oncology
LEG1	EPHA1	ITGB1BP1
LGALS3	EPHX2	KRT18
LGMN	EVI5	KRT5
LILRA4	FARSA	LAMB1
LPA	FCGR2A	LBP
LRG1	FCRLB	LEG1
LTB	FGFBP1	LPA
LY9	GAST	LRG1
LZTFL1	GCLM	LY9
MBL2	GGCT	LYPD8
MFAP5	GKN1	MBL2
MRPL58	GLRX	MMP10
MUC16	GMPR	NEDD9
MYCBP2	HAVCR1	NPTN
NDRG1	ICA1	NPTX2
NOP56	IKBKG	NUB1
NPTXR	IL-10	PAFAH2
NTRK2	INPP5D	PALM3
OLR1	IRAK4	PGA4
PDE5A	KIAA0319	PLA2G2A
PFKFB2	KLK13	PPIE
PLA2G4A	KLK8	PRR4
PPP1R12A	LARP1	PTPRK
PTH	LRTM2	PTPRM
PTPRK	LYPLA2	PVR
PTPRN2	LZTFL1	REG3A
PZP	MFAP3L	RELT
RELT	MNDA	RGL2
RGMA	MYCBP2	S100A13
RNASE4	NCF2	SAA4
S100A12	NFE2	SCIN
SAA4	NFKB1	SCT
SCRIB_Neurology_II	NOP56	SERPINA7

SCT	NPTN	SFRP4
SLAMF1	NPTXR	SOWAHA
SLITRK1	NRTN	SPON2
SPON1	NT5C3A	SRPX
SRP14	NTRK2	SUSD4
STAT5B	OTUD6B	TCP11
SUSD4	PALM3	TIGIT
SV2A	PDAP1	TNFRSF6B
TCP11	PDE5A	TNFRSF8
TET2	PFKFB2	TRIM25
THBS4	PIK3AP1	TRIM40
TIGAR	PLA2G2A	TSPAN1
TIGIT	PLPBP	TSPAN7
TNR	PPP1R12A	VMO1
TPD52L2	PPP1R2	VWA1
TPP1	PRKRA	WFIKKN1
TRIM40	PSAPL1	
TSC1	PTRHD1	
UNC5D	RAB44	
VASP	RASSF2	
WFIKKN1	REG3A	
	RSPO1	
	RSPO3	
	S100A4	
	SAMD9L	
	SDHB	
	SERPINI2	
	SFRP1	
	SH3GLB2	
	SHBG	
	SKAP1	
	SLITRK1	
	SNX9	
	SPINK4	
	SPOCK1	
	STAMBP	
	STAT5B	
	TANK	
	TAX1BP1	
	TBC1D17	
	TBCA	
	TBCC	
	TNFSF12	
	TNIP1	
	TOP2B	

TRAF3
TRIM25
TSPAN1
TXN
TXNDC9
UNC5D
USP25
VASP
VTA1
YARS1

Supplemental Table 10. Top 10 signigicant enriched pathways identified in proteomics

Pathway	Number of total proteins within the pathway	Number of significant differentially expressed proteins within the pathway	P value	Q value
Senescence-Associated Secretory Phenotype (SASP)	17	8	8.15E-08	0.00017
Interleukin-10 signaling	42	10	3.17E-06	0.00326
Cellular Senescence	28	8	7.15E-06	0.00490
MAPK1 (ERK2) activation	6	4	3.04E-05	0.01249
Interleukin-6 signaling	6	4	3.04E-05	0.01249
Interleukin-4 and Interleukin-13 signaling	57	10	5.53E-05	0.01893
RAF-independent MAPK1/3 activation	8	4	1.33E-04	0.02740
MAPK3 (ERK1) activation	8	4	1.33E-04	0.02740
CD163 mediating an anti-inflammatory response	8	4	1.33E-04	0.02740
ATF4 activates genes in response to endoplasmic reticulum stress	8	4	1.33E-04	0.02740

Supplemental Table 11. List of LPC species and cytokines included in the association

LPC species	Cytokine
LPC(16:0)	CCL2
LPC(16:1)	CCL3
LPC(17:0)	CCL4
LPC(18:0)	CCL5
LPC(18:1)	CCL7
LPC(18:2)	CCL8
LPC(20:3)	CCL11
LPC(20:4)	CCL13
	CCL14
	CCL15
	CCL16
	CCL17
	CCL18
	CCL19
	CCL20
	CCL21
	CCL22
	CCL23
	CCL24
	CCL25
	CCL26
	CCL27
	CCL28
	CSF1
	CSF2
	CSF3
	CX3CL1
	CXCL1
	CXCL3
	CXCL5
	CXCL6
	CXCL10
	CXCL11
	CXCL12
	CXCL13
	CXCL14
	CXCL16
	CXCL17
	CXCL8-cardiometabolic
	CXCL8-inflammation
	CXCL8-neurology
	CXCL8-oncology
	CXCL9
	CYTL1
	FLT3LG
	IFNG
	IFNL1
	IFNL2
	IFNW1
	IL-1A
	IL-1B
	IL-1RN

IL-2
IL-3
IL-4
IL-5
IL-6-cardiometabolic
IL-6-inflammation
IL-6-neurology
IL-6-oncology
IL-7
IL-9
IL-10
IL-11
IL-12A_IL-12B
IL-12B
IL-13
IL-15
IL-16
IL-17A
IL-17C
IL-17D
IL-17F
IL-18
IL-19
IL-20
IL-22
IL-24
IL-25
IL-27A
IL-31
IL-32
IL-33
IL-34
IL-36A
IL-36G
LECT2
LIF
OSM
PF4
PPBP
SCGB1A1
SPP1
TAFA5
TGFB1
TGFB2
TNF-cardiometabolic
TNF-inflammation
TNF-neurology
TNF-oncology
TNFSF8
TNFSF9
TNFSF10
TNFSF11
TNFSF12
TNFSF13
TNFSF13B
TNFSF14

	XCL1

CXCL8, IL-6 and TNF were measured in 4 different panels and are considered distinct proteins.

Supplemental Table 12. Baseline characteristics of 16 subjects with VHR ALL

	Controls			AAP cases		
	**N**	**%**	**Median (range)**	**N**	**%**	**Median (range)**
**Total no. of subjects**	7			9		
**Age at diagnosis, y**			8 (1~15)			6 (1 ~15)
1 ~ 10	5	71.4		7	77.8	
10 ~ 15	2	28.6		2	22.2	
**Sex**						
Female	2	28.6		4	44.4	
Male	5	71.4		5	55.6	
